# Supplementary material for: Adjusting the energy of interfacial states in organic photovoltaics for maximum efficiency
Source: Nat Commun. 2021 Mar 19;12:1772. doi: 10.1038/s41467-021-22032-3 (PMC7979693; doi:10.1038/s41467-021-22032-3)
Supplement: Supplementary file 1 — Supplementary Information [file 41467_2021_22032_MOESM1_ESM.pdf]

Supplementary information:

Adjusting the Energy of Interfacial States in Organic Photovoltaics for Maximum Efficiency

Nicola Gasparini*[1,2,+], Franco V.A. Camargo[3,+], Stefan Frühwald[4], Tetsuhiko Nagahara[3,5], Andrej Classen[2], Steffen Roland[6], Andrew Wadsworth[7], Vasilis G. Gregoriou[8,9], Christos Chochos[8,10], Dieter Neher[6], Michael Salvador[11], Derya Baran[11], Iain McCulloch[7,11], Andreas Görling[4], Larry Lüer*[2], Giulio Cerullo[3], Christoph J. Brabec*[2,12,13]

[1]Department of Chemistry and Centre for Plastic Electronics, Imperial College London, SW72AZ, UK

[2]Institute of Materials for Electronics and Energy Technology (I-MEET), Friedrich Alexander-University Erlangen-Nuremberg, Martensstraße 7, 91058 Erlangen, Germany

[3]IFN-CNR, Dipartimento di Fisica, Piazza Leonardo da Vinci 32, 20133 Milano, Italy

[4]Department of Chemistry and Pharmacy, Friedrich Alexander-University Erlangen-Nuremberg, Egerlandstr. 3 91058 Erlangen, Germany

[5]Department of Chemistry and Materials Technology, Kyoto Institute of Technology, Matsugasaki, Kyoto 6068585, Japan

[6]Institut für Physik und Astronomie Physik weicher MaterieUniversity of Potsdam14476 Potsdam, Germany

[7]Department of Chemistry, Chemistry Research Laboratory, University of Oxford, Oxford, OX1 3TA, UK

[8]Advent Technologies SA, Stadiou Street, Platani, Rio, Patras 26504, Greece

[9]National Hellenic Research Foundation, 48 Vassileos Constantinou Avenue, Athens, 11635, Greece

[10]Institute of Chemical Biology, National Hellenic Research Foundation, 48 Vassileos Constantinou Avenue, Athens, 11635, Greece

[11]King Abdullah University of Science and Technology (KAUST), Division of Physical Sciences and Engineering (PSE), KAUST Solar Center (KSC), Thuwal, 23955, Saudi Arabia

[12]Bavarian Center for Applied Energy Research (ZAE Bayern), Haberstrasse 2a, 91058 Erlangen, Germany

[13]Helmholtz-Institute Erlangen-Nürnberg (HI ERN), Erlangen, Germany.

[+]These authors contributed equally to this work: Nicola Gasparini, Franco V.A. Camargo

*Corresponding authors: N. Gasparini (n.gasparini@imperial.ac.uk), L. Lüer (larry.lueer@fau.de), C.J. Brabec (christoph.brabec@fau.de)

TABLE OF CONTENTS:

SUPPLEMENTARY NOTE 1: Modeling of ground state absorption spectra

SUPPLEMENTARY NOTE 2: Modeling of transient absorption data

SUPPLEMENTARY NOTE 3: Determination of the effective CT energy

SUPPLEMENTARY NOTE 4: Quantum-Chemical calculations

SUPPLEMENTARY NOTE 5: Electrostatic calculations

SUPPLEMENTARY NOTE 6: Transient absorption after pumping with narrowband pulses

SUPPLEMENTARY NOTE 7: Photoluminescence transients

SUPPLEMENTARY NOTE 8: Organic solar cells fabrication and characterizations

SUPPLEMENTARY NOTE 9: Estimation of pump-induced relative excitation density

SUPPLEMENTARY NOTE 10: Appendix. Procedure of spectral decomposition into contributions from physical states

SUPPLEMENTARY NOTE 1: Modeling of ground state absorption spectra

Modeling of ground state absorption (GA) and photoluminescence (PL) spectra is an important prerequisite for a correct interpretation of photobleaching (PB) and stimulated emission (SE) features, respectively, in transient absorption (TA) data. Supplementary Fig. 1 shows PL and GA spectra of as-deposited WF3 thin films (solid lines). Fits are given as dashed lines. The PL spectra can be modeled by a single effective vibronic progression of Gaussians with a (00) transition at 1.692 eV, an energy of the effective vibronic progression of 157 meV, and an effective Huang-Rhys (HR) factor of 0.83, which might be slightly exaggerated due to reabsorption effects.

For the modeling of GA in WF3, we found that we can only obtain a good fit by assuming the simultaneous presence of an amorphous and an ordered phase, both showing similar HR factors as PL. Assuming a single phase requires an HR factor of 1.6, in stark contrast with the values from PL. The ordered phase is redshifted against the amorphous phase by about 180 meV and is characterized by narrower Gaussian bands.

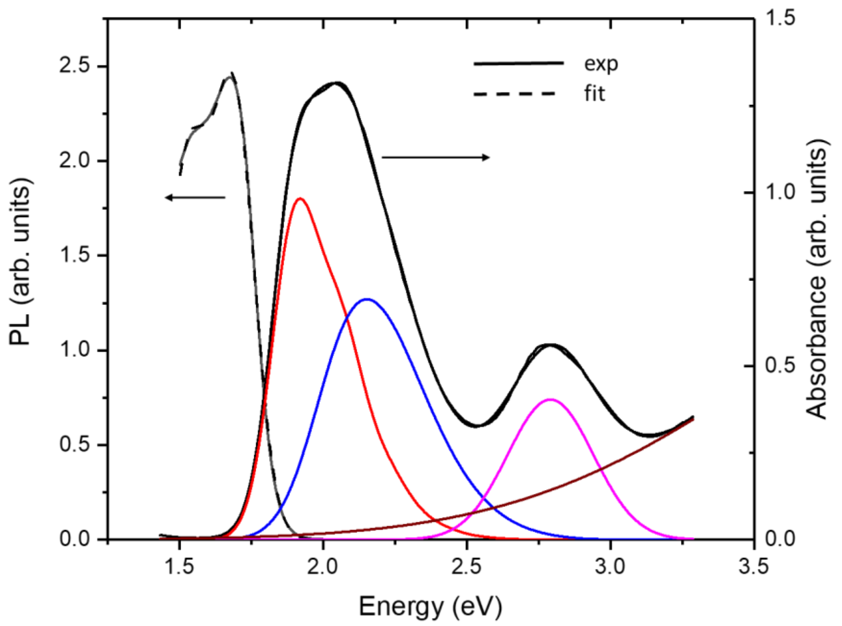

Supplementary Figure 1. Photoluminescence (PL, left solid black line) and ground state absorption (GA, right solid black line) spectra of a pristine as-deposited WF3 film and multi-band fits (dashed black lines). The individual contributions to the fit of GA are: the lowest energetic optical transitions of the ordered and the amorphous phase (red and blue, respectively), the next higher optical transition of both phases (pink) and the contribution of scattering (brown).

GA and PL spectra of annealed O-IDTBR are shown in Supplementary Fig. 2. The optical absorption bands are superposed with strong scattering contributions pointing to the presence of grain boundaries with a domain size on the order of the wavelength of the irradiated light (Mie scattering). This can be deduced from the fact that assuming a monotonous (Rayleigh, particles much smaller than the wavelength) or constant (particles much bigger than the wavelength) scattering function, the GA spectrum cannot be reproduced assuming vibronic progressions of Voigt or Gaussian band

shapes. Only a scattering function with a maximum in the vicinity of the lowest energetic absorption band yields a good fit assuming reasonable parameters for the HR factor and the band widths. We note however that the uncertainty of HR and the bandwidths is substantial given the uncertainty of the superposed scattering function; only the band center and the energy of the effective progression can be given precisely (1.652 eV and 240 meV, respectively).

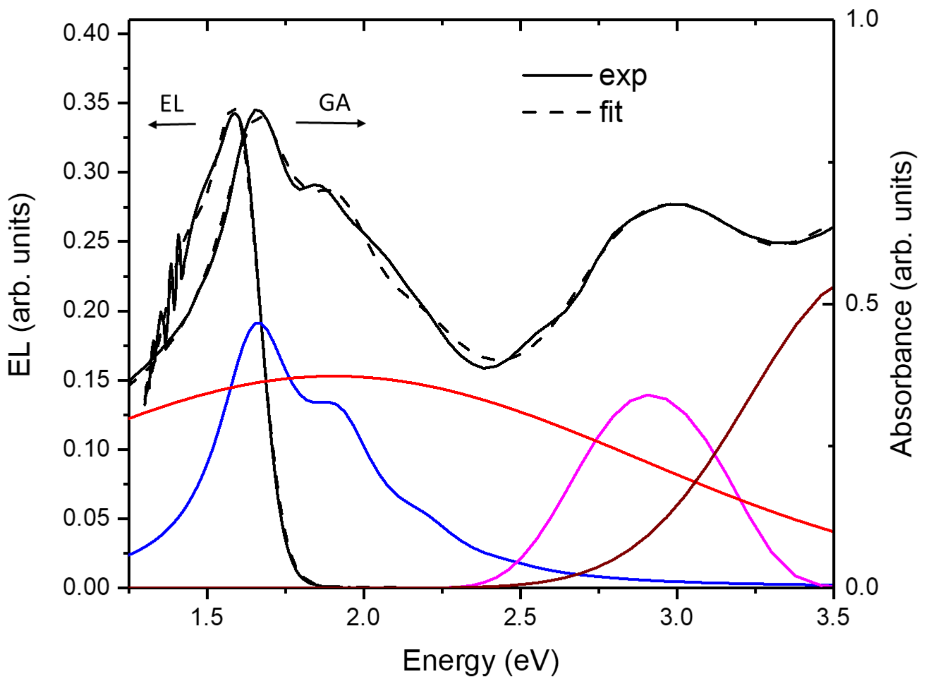

Supplementary Figure 2. Electroluminescence (EL, left black solid line) and GA spectra (right black solid line) of an annealed O-IDTBR film (black lines) and multi-band fits (dashed lines). The individual contributions to the fit of GA are: the lowest energetic optical transition (blue)and the next higher optical transition (pink) The strong background of the GA spectrum was ascribed to reflection and Mie type scattering losses, qualitatively modeled by broad Gaussians (red and brown)

In Supplementary Fig. 3, we show a GA spectrum of the blend (grey solid line). It can be fitted (black dashed line) quite well by a superposition of contributions from the pure as-deposited WF3 film and the annealed O-IDTBR film (red and blue lines, respectively). As Supplementary Table 1 shows, the center energies for both pure materials and the band widths had to be slightly adapted for a good fit.

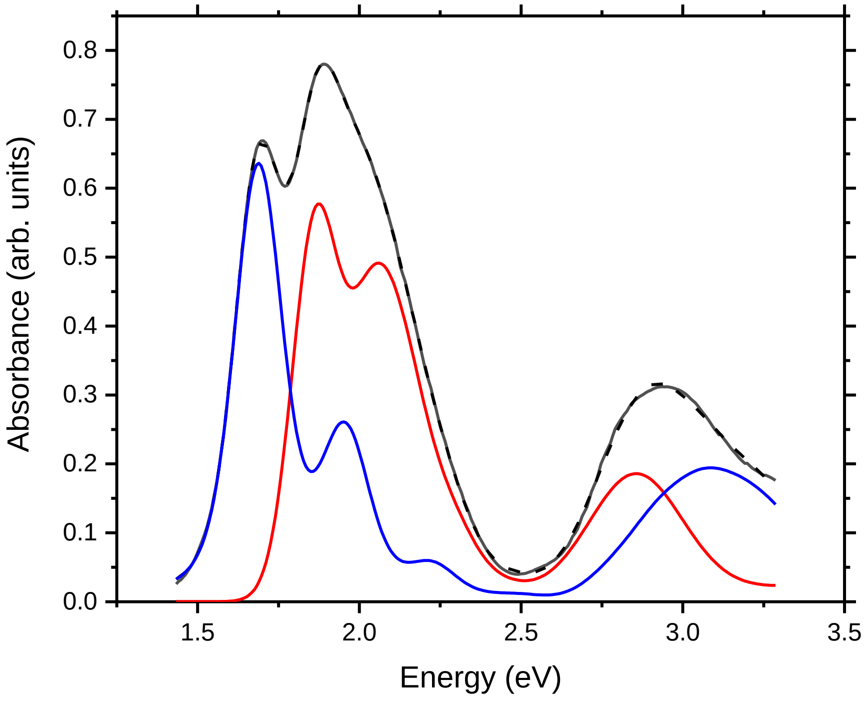

Supplementary Figure 3. GA spectrum of the WF3:O-IDTBR blend (black solid lines) and a fit (black dashed line) assuming a superposition from pure WF3 and pure O-IDTBR (red and blue, respectively).

SUPPLEMENTARY NOTE 2: Modeling of transient absorption data

Transient absorption (TA) spectroscopy shows optical probes from various photoexcited states in a single experiment, thus making it an ideal tool to study complex photoexcitation dynamics. However, organic materials typically have broad TA bands, which is due to disorder and vibronic coupling. This generally leads to spectral congestion, which must be treated by matrix decomposition techniques coupled with nonlinear optimization schemes in order to obtain time-resolved concentrations of all contributing photoexcitations individually.[i] Our approach has been detailed in a previous publication.[ii] In short, we typically measure time-resolved TA spectra (two-dimensional matrices as function of probe photon energy ω and pump-probe delay time *t*) varying a third parameter such as the pump intensity *J*. Following Lambert-Beer's Law, the experimental transient absorption A_{exp} is given by:

$$A_{exp} = \sum_{m=1}^{N} \sigma_m(\omega_m, \chi_m) \cdot \mathrm{s}_m(t), \qquad \text{(S1)}$$

where $s_m(t)$is the time-resolved area density of state m, and σ_i is the absorption cross-section of that state, which depends on the probe energy ω_{pr} and follows a spectral model, for example, a combination of Gaussian bands whose parameters (relative strength, width, center, etc) are given in terms of the vector of hyperparameters χ_m. Goal is to find $\mathrm{s}_m(t)$ for all interacting states. To this end, we perform a singular value decomposition (SVD) yielding characteristic spectra and dynamics. We retain only the signal-related characteristic spectra and corresponding dynamics, given by the $U_{S,}$ and V_S matrices, respectively, rejecting noise. The characteristic spectra U_S are related to the matrix σ of absorption cross-sections via a rotation matrix R by

$$U_S \cdot R = \sigma, \qquad \text{(S2)}$$

The rotation matrix R is obtained by Moore-Penrose pseudo inversion while varying the hyperparameters χ of the cross-section spectra in σ in a nonlinear optimization loop. Once the rotation matrix is obtained, the desired photoexcitation dynamics is obtained by

$$s = R^{-1} \cdot S_S \cdot V_S \, , \tag{S3}$$

where S_S is the set of signal related singular values, and the matrix s is composed of the individual $s_m(t)$ in eq. S1. A detailed derivation is given in the Appendix of this Supporting Information.

We use as much experimental evidence as possible to reduce the number of free hyperparameters in χ. For example, for each state we train the hyperparameters using pure films. Then we use S2 is applied to predict $s_m(t)$ for the blends, keeping most of the hyperparameters to those found in the pure films. If we allow hyperparameters to vary, then it is because of a clearly described physical reason. In the Appendix of this Supporting Information, we give the full theory and the rules which hyperparameters are allowed to be optimized.

2a Training of spectral models: pure WF3 and o-IDTBR films

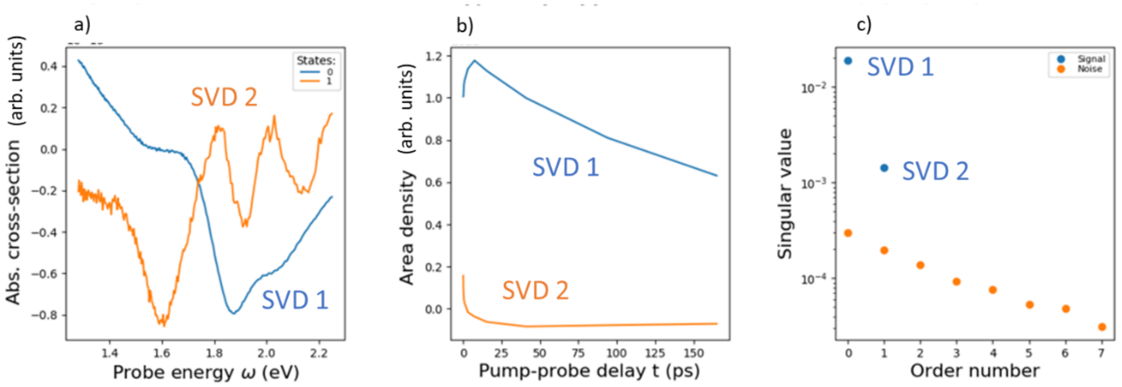

Supplementary Figure 4. Singular value decomposition (SVD) of a time-resolved TA spectrum (10 fs broadband pulses, parallel polarization of pump and probe pulses) of an as-deposited WF3 film, using two signal-related singular value. a) Basis spectra, b) dynamics, c) singular values (blue symbols: signal related; orange: noise-related).

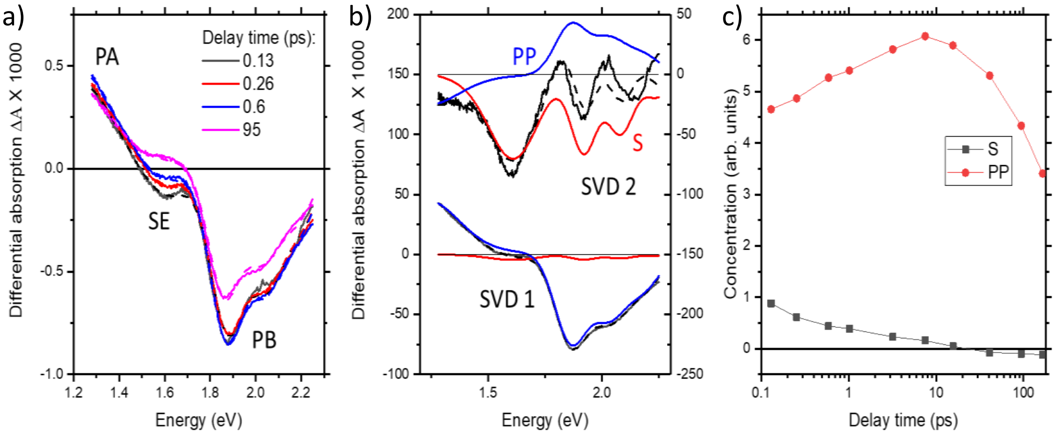

Supplementary Figure 5. a) Time-resolved transient absorption (TA) spectra of an as-deposited WF3 film after pumping with 10 fs broadband pulses (solid lines, different colors referring to different delay times, as given). Dashed lines are fits obtained by summing up the dot products between spectra and concentration for each contributing state. b) The two strongest basis states of an SVD of the time-resolved TA spectrum in Supplementary Figure 5a (black solid lines) and fits (dashed black lines) obtained by a

weighted superposition of simulated spectra of emissive singlet states (S, red solid lines) and polaron pairs (PP, blue solid lines). c) Resulting time-resolved concentrations of S and PP states, according to eq. S14.

In Supplementary Fig. 4, we show a SVD of a time-resolved TA spectrum (10 fs broadband pulses, parallel polarization of pump and probe pulses) of an as-deposited WF3 film. Prior to performing the SVD analysis, the spectra have been logarithmically binned along the time axis into 12 bins associated with an effective delay time each (X axis in panel b). The singular values are shown in panel c in descending order, each singular value associated with a column vector (basis spectra) and a row vector (dynamics). As shown in panel c, there are two singular values clearly offset in strength against the other ones, which means that the associated basis spectra and dynamics (shown in panel a and b, respectively) will capture nearly all spectral and dynamic information. The rest of the singular values will be dominated by noise, however, they might still contain some spectro-kinetic information, such as spectral migration and line narrowing effects. In the present case, it is acceptable to ignore these effects, because the strongest noise-related singular value is a factor of 5 weaker than the weakest signal-related singular value, see panel c.

In Supplementary Fig. 5, we show time-resolved TA spectra of a WF3 film (solid lines of different color according to delay times, as given in legend). The TA spectra exhibit transient photobleach (PB) features around 1.9 eV, in the region of GA, an a broad, a structurelessPA band peaking in the near infrared, outside of the spectral range of our probe pulse. For delay times below 1 ps, a second band of formally negative differential absorption is present around 1.6 eV. As this is outside of the range of GA, it can only be associated with SE. However, the spectral position of the SE band in Supplementary

Fig. 5a does not exactly agree with that of PL in Supplementary Fig. 1, which peaks at around 1.7 eV. This might be explained by emission from a vibrationally hot state.

In Supplementary Fig. 6, we show an SVD of a time-resolved TA spectrum of an annealed film of O-IDTBR. From the list of singular values (Supplementary Fig. 6c) we conclude that there is one dominant state SVD1 which we consider as signal related. It is characterized by a strong negative peak around 1.68 eV surrounded by weaker peaks at 1.4 and 1.9 eV, suggesting a strongly emissive state in which both PB and SE are superposed. Indeed, in Supplementary Fig 7, we show that SVD1 can be approximately reproduced (Supplementary Fig. 7a) by a weighted superposition of inverted replicas of GA and PL from Supplementary Fig. 2, adding two PA bands (Supplementary Fig. 7b). This spectral model yields an acceptable fit of the original time-resolved TA spectrum over the entire temporal scale, see Supplementary Fig. S7c.

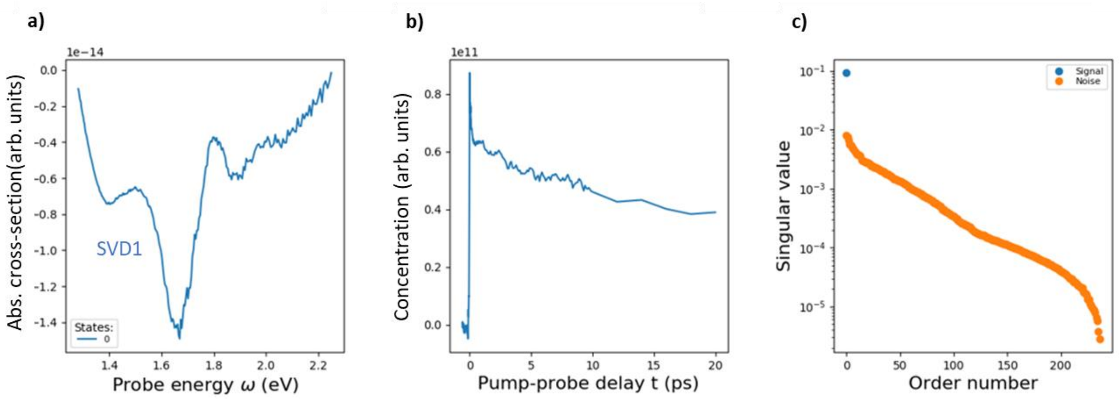

Supplementary Figure 6. SVD of a time-resolved TA spectrum (10 fs broadband pulses, parallel polarization of pump and probe pulses) of an annealed O-IDTBR film, using a single signal-related singular value. a) Absorption cross-section, b) concentration, c) singular values (blue symbols: signal related; orange: noise-related)

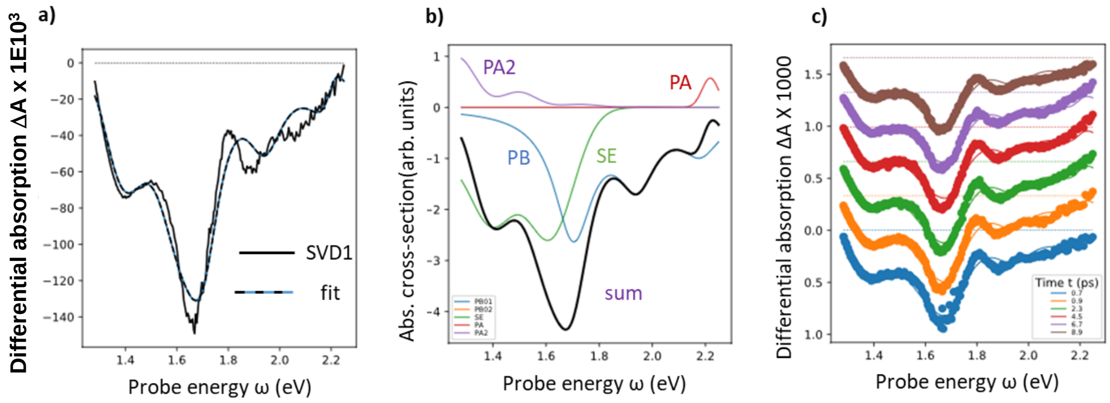

Supplementary Figure 7. Spectral reproduction of SVD1 in Supplementary Figure 7a by a superposition of PB, SE (derived from GA and PL, respectively, in Supplementary Figure 7b-c) and singlet excited state absorption, PA and PA2. Parameters are given in Supplementary Table 1.

2b Spectral modeling of WF3:o-IDTBR blends

In Supplementary Fig. 8, we show an SVD of a time-resolved TA spectrum of the WF3:O-IDTBR blend film. In panel c, we see that there are 3 singular values above the noise-related continuum, all three of them showing clear spectra and dynamics (Supplementary Fig. 8 a and b, respectively). In Supplementary Fig. 9, we performed spectral modeling of the basis spectra of Supplementary Fig. 8a to obtain the photoexcitation dynamics from the SVD dynamics in Supplementary Fig. 8b. While the samples in Supplementary Figs. 5 and 7 are made from the pure donor and acceptor material, respectively, and thus support neutral excited states, we expect the formation of charged states in the blends, because of the presence of the donor-acceptor interface. As we explain by discussing Supplementary Fig. 11, these charged states will in general have different excited state absorption bands than the neutral excitations. However, they share the same PB and show no SE. In the absence of further effects, the PB region in

Supplementary Fig. 9 is therefore expected to be a weighted superposition from the PB regions of WF3 and O-IDTBR singlets (Supplementary Fig. 5a and 7a, respectively).

However, Supplementary Fig. 9a clearly shows that this is not the case. Only upon introduction of first and second derivatives of the GA spectra of O-IDTBR and the ordered phase of WF3 could we find an acceptable fit of all three SVD spectra. Such derivatives are known to stem from transient Stark effects ('electroabsorption', EA) due to static electric fields caused by charged states acting upon neighboring neutral molecules. Hence, the overall blend signal is composed from:

a) LEs of each material, which includes the corresponding PB, SE and PA;
b) charge transfer (CT) and charge separated (CS) states, which include PB of both WF3 and O-IDTBR, and also their own PA;
c) EA, which is an indirect signature of the CT/CS states, arising from a Stark shift of molecules in vicinity of the separated charges that feel the resulting static electric field. As stated above, this contribution corresponds to derivative bands of both material's PB.

The resulting dynamics are given in Supplementary Fig. 9bc; it is obvious that the EA-related features decay more slowly than the population-related features CT+CS and LE.

A relative increase of the EA contribution with respect to the population contribution has been ascribed to charge separation.[iii] The dynamics of this relative increase is therefore a measure of the initial charge separation kinetics, i.e., from an interfacially bound CT state to state where at least one of the charged states is no longer at the interface. With these spectral models, we achieve a good reproduction of the original TA spectra at all delay times, considering that all spectral shapes are derived from other optical probes and no new bands have been introduced (Supplementary Fig. 10 a,b).

For the determination of the spectral model of CT+CS, we produced an SVD of the TA spectrum for t>100 ps, where we know from PL transients that all LE(O-IDTBR) states have decayed. The remaining PB of O-IDTBR can thus be ascribed to the charged state of the O-IDTBR, which allows us to determine the weight ratio of the PB contributions from WF3 and O-IDTBR in CT+CS. This ratio is then frozen for the spectral modeling of the full TA spectrum, thus forcing the LE(O-IDTBR) contribution to zero, as shown in Supplementary Fig. 9c.

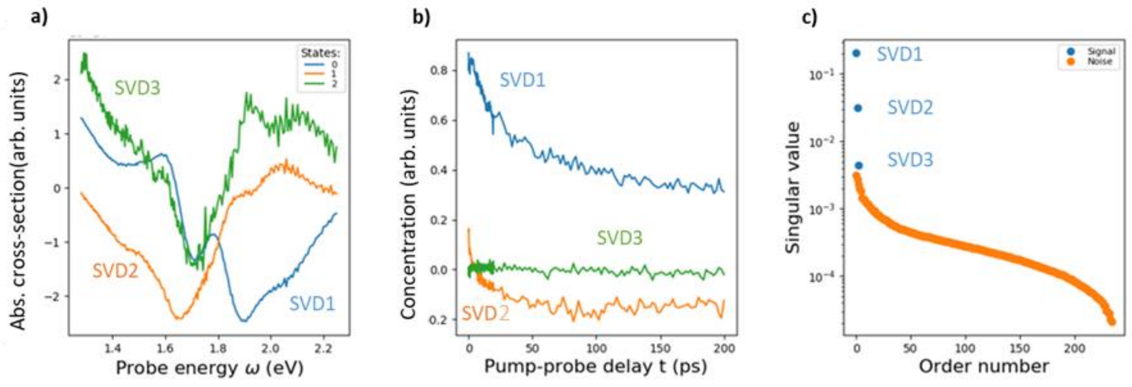

Supplementary Figure 8. SVD of a time-resolved TA spectrum (10 fs broadband pulses, parallel polarization of pump and probe pulses) of the WF3:O-IDTBR blend, using three signal-related states. a) Basis spectra, b) dynamics, c) singular values (blue symbols: signal related; orange: noise-related)

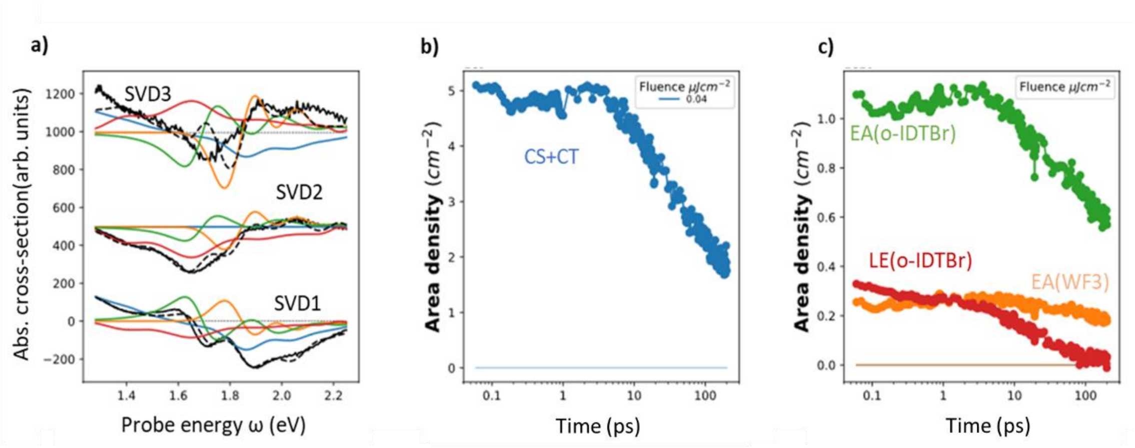

Supplementary Figure 9. Spectral reproduction of SVD 1, 2, and 3, from Supplementary Fig. 7, by a weighted superposition of contributions from charge separated and interfacial charge transfer states (CS and CT, respectively), local excitations in the O-IDTBR phase (LE O-IDTBR, spectral model from Supplementary Fig. 6), and electroabsorption (EA) contributions (weighted superposition of first and second derivatives of the spectral models for GA of the ordered phase of WF3 and GA of annealed O-IDTBR, see Supplementary Figs. 1 and 2, respectively). For the construction of the spectral model of CS+CT, see text.

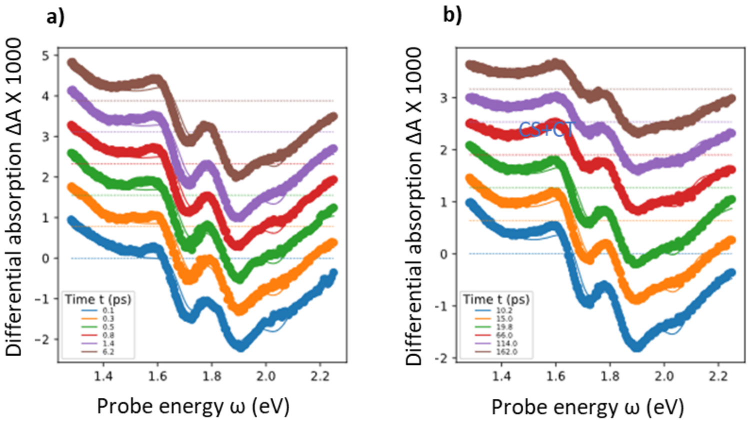

Supplementary Figure 10: Reproduction of the original time-resolved TA spectrum of the WF3:O-IDTBR blend (thick lines) by a matrix multiplication of the colored spectra

in Supplementary Fig.10a with the colored dynamics in Supplementary Fig- 10b,c. Pump-probe delay times are given in the inset.

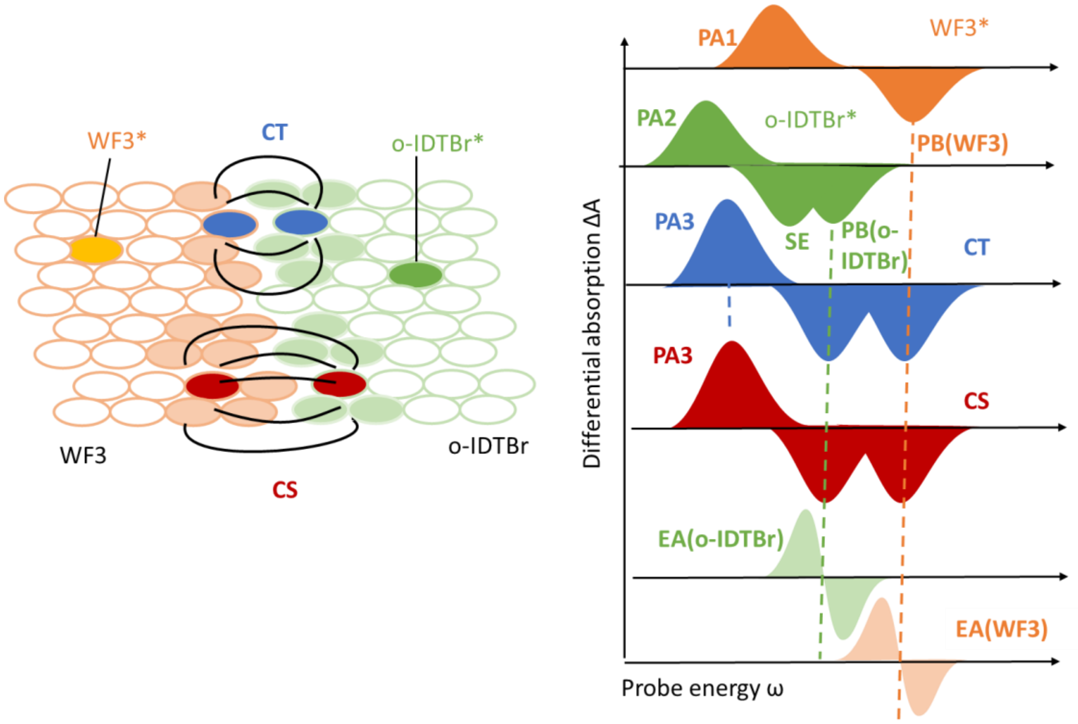

Supplementary Figure 11: left: schematic representation of states contributing to the TA spectra in Supplementary Figure 9-10; explanation of respective optical probes. Shapes with no fill indicate molecules in the ground state in the absence of static electric fields; colored shapes are molecules in the electronically excited state (O-IDTBR*: emissive exciton of the acceptor; WF3*: polaron pair [symmetric charge transfer state] of the donor) or carrying a net charge; CT: interfacial charge transfer state; CS: charge separated state, where at least one charged molecule is not at the interface. Softly colored shapes are molecules in the electronic ground state but exposed to field lines of a charge pair, exhibiting a Stark shift of the fundamental electronic transition. We draw field lines only where the field vectors of the individual charges superpose constructively. Right: expected contributions to the TA spectra of these states, namely

PA (photoinduced absorption), PB (photobleach), SE (stimulated emission), and EA (electroabsorption). Identical bands occurring in several states are connected by dashed vertical lines. For further explanations, see text.

In Supplementary Fig. 11, we give a schematic representation of the states contributing to the TA spectra in Supplementary Figs. 9 and 10 and explain their specific optical probes. We start our discussion with the formula for the calculation of the differential absorption,

$$\Delta A(\omega,t) = A_{pumpon}(\omega,t) - A_{pumpoff}(\omega,t) = d \cdot \sum_i \sigma_i(\omega) \cdot \Delta c_i(t); i \in. \quad \text{(S4)}$$

Here, d is the film thickness, σ is the absorption cross-section, ω is the probe photon energy, D^0 and A^0 are donor and acceptor molecules, respectively, in the ground state and not exposed to a static electric field, D^F and A^F ground states exposed to a static electric field, D* is a symmetric charge transfer exciton in the donor phase (non-emissive and not carrying net charges) and A* are emissive excitons in the acceptor phase, D^+ are positive charges in the donor phase and A^- are negative charges in the acceptor phase.

We further assume that at our repetition rate of 1 kHz, all pump-induced excited states have decayed back to the ground state (verified by the fact that there is vanishing TA signal for negative delay times). Furthermore, there is no external electric field. Then, only D^0 and A^0 will contribute to $A_{\text{pump off}}$, the concentration of all other states being zero. Therefore,

$$\Delta c_j = -\Delta c_{A^0}; j\varepsilon, \quad \text{(S5)}$$

which means that any state produced in the donor phase will, apart from the specific photoinduced absorption bands PA, also produce a ground state photobleach PB caused by the reduction of the concentration of ground state molecules. The same is true for the acceptor phase. Hence, PA bands are specific for the kind of photoexcitation but not for the phase, while PB bands are specific for the phase in which the excited states dwell but not for the kind of photoexcitation.

Turning now to the right part of Supplementary Fig. 11,

- all the electronically excited states WF3*, O-IDTBR*, or charged states CT and CS, will produce an individual unique set of PA bands (shown at lower probe energy). It is however possible, that due to the intramolecular CT character of low energetic transitions in low- bandgap moieties, these bands are relatively close together so that they often cannot be safely distinguished.
- all the electronically excited states in the donor phase must exhibit a PB band which is the inverted replica of the GA of that phase (ignoring hole burning and spectral migration)
- same for all excited states in the acceptor phase.

This means that, CS and CT (and hence, their time-resolved populations) cannot be distinguished by looking at the PB. Both will produce PB in both phases. However, being charged these states cause static electric fields that red shift the *ground state absorption (GA)* in neighboring non-excited molecules (Stark effect). Since this shift is much smaller than the bandwidth, the resulting TA spectra can be approximated by first derivatives of the GA. A differential absorption upon modulation of static electric fields

is called electroabsorption (EA); in our experiment we modulate the field by modulating the number of charged pairs causing it.

The number of molecules situated between the charged pair, where the field lines superpose constructively, is larger in a CS than in a CT state. This means that although we can only measure the sum of the populations of CS+CT (by virtue of their common PB), we can get an indication of their relative weight (and hence the transition from CT to CS) by comparing the evolution of the intensity of the EA bands to that of the PB bands (see point E at the end of this ESI for a simple electrostatic simulation). In order to distinguish them, we make use of the fact that the first derivative of a band has zero integral. (see dashed vertical lines in Supplementary Figure 11). Hence, if in the TA spectrum, the spectral weight of the negative and the positive lobe of the first derivative contribution are not equal, we know that the spectral region is superposed with PB. This distinction is systematically exploited by the matrix decomposition technique that we deploy.

SUPPLEMENTARY NOTE 3: Determination of the effective CT energy

We use a multi-objective optimization to reproduce the crucial observations of the main text (an EQE exceeding 80%, a charge separation time of 40 ps, and an EL spectrum which is indistinguishable from the pure LE spectrum) by the simplest possible rate equation model. Using the known overall driving force (difference between the LE and CS energies) and the experimental or calculated LE and CT deactivation rates, this allows us to determine the effective CT energy, relative to that of the LE and CS energies, as well as the rate constants for the two equilibration reactions (LE ⬄ CT, CT⬄ CS).

Given the notion of an equilibrium between LE and CT states[iv] and between CT and CS states,[v] the simplest ordinary differential equation (ODE) scheme is as follows (see Supplementary Figure 12):

$$d\,(\mathrm{LE})/dt = G_{pe} - \left(k_r + k_{nr} + k_{\mathrm{LE},CT}\right) \cdot \mathrm{LE} + k_{CT,\mathrm{LE}} \cdot CT \quad \text{(S6)}$$

$$d\,(CT)/dt = k_{\mathrm{LE},CT} \cdot \mathrm{LE} - \left(k_{r,CT} + k_{nr,CT} + k_{CT,CS} + k_{CT,\mathrm{LE}}\right) * CT + k_{CS,CT} \cdot CS + k_L \cdot CS^2 \quad \text{(S7)}$$

$$d\,(CS)/dt = G_{inj} + k_{CT,CS} \cdot CT - k_{CS,CT} \cdot CS - k_{extr} \cdot CS - k_L \cdot CS^2 \quad \text{(S8)}$$

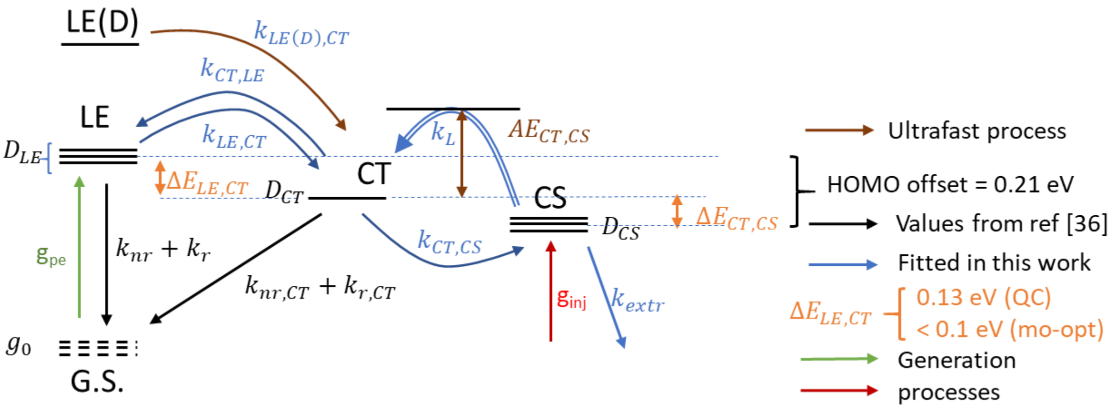

Supplementary Figure 12. Pictorial representation of the rate equation scheme. The green arrow refers to optical excitation while the red arrow refers to electrical injection. Single arrows refer to first-order processes while the double arrow refers to bimolecular Langevin recombination of charge carriers. Black arrows refer to processes whose rate constants are known in the present system. The degeneracy of the states is given by g0..g3. Dashed states have not been included explicitly into the rate equation scheme.

In the ODE system, k_r and k_{nr} are the radiative and non-radiative rate constants of LE deactivation, $k_{r,CT}$ and $knr_{,CT}$ are the respective deactivation rate constants of interfacial CTstates, $k_{LE,CT}$ and $k_{CT,LE}$ are the forward and backward, respectively, rate constants for the equilibrium LE $\Leftrightarrow$CT, $k_{CT,CS}$ and k_L are the forward and backward, respectively, rate constants of the equilibrium CT $\Leftrightarrow$CS, where CS is a charge separated state with the positive charge in the donor phase and the negative one in the acceptor phase, k_{extr} is the rate constant for charge extraction, here assumed first order thus ignoring drift and diffusion phenomena. Generation of LE states occurs through generation function g_{pe}, where "pe" stands for photoexcitation, which can be either a delta pulse (for transient experiments) or a constant (for continuous wave illumination), while injection of CS states occurs with generation function g_{inj}, which is a constant.

We use the published values of k_r, k_{nr}, $k_{r,CT}$ and $k_{nr,CT}$ and the overall driving force given by the HOMO offset of 0.21 eV, see Supplementary Table 1. Therefore, the unknown parameters are the effective CT energy, the rate constants controlling both equilibria, and the extraction rate constant. As the forward and backward rate constants in both equilibria are linked by the Boltzmann equilibrium constant, the total number of degrees of freedom of the problem is four.

For the multi-objective optimization, we solve the ODE system (S6-S8) iteratively varying the four degrees of freedom and setting the initial conditions in order to model correctly the following three objectives:

(1) To simulate EQE, we calculate the stationary state of the ODE system (S6-S8) by letting g_{pe}=const and setting the left sides zero, and calculate IQE as

$$IQE = \frac{d(e^{-}+h^{+})/dt}{L_{\mathrm{abs}}} = CS_{st} \cdot k_{extr} \cdot d_{AL}/g_{\mathrm{pe}}, \tag{S9}$$

where e^- and h^+ is the area flux of extracted electrons and holes, respectively, CS_{st} is the stationary concentration of charge separated states under an absorbed stationary illumination flux g_{pe}, which is set to one sun under AM1.5 conditions and a bandgap of 1.65 eV, considering an effective optical density of 0.6 due to above-bandgap transmission, reflection and parasitic absorption losses, and d_{AL} is the active layer thickness.

(2) The observed EL spectra are calculated as the sum of the individual contributions from CT and LE states, EL_{LE} and EL_{CT}, respectively. For this experiment, injection is modeled via setting $g_{inj}(t)$ = const in eq. S8 and solving for stationary condition:

$$EL(\omega_{pr}) = EL_{\mathrm{LE}}(\omega_{pr}) + EL_{\mathrm{CT}}(\omega_{pr})$$
$$= r \cdot \left(\mathrm{LE}_{st} \cdot k_r \cdot \sigma_{\mathrm{PL,LE}}(\omega_{pr}) + CT_{st} \cdot k_{r,CT} \cdot \sigma_{\mathrm{PL,CT}}(\omega_{pr})\right)$$

(S10)

Herein, $\mathrm{LE_{st}}$ and $\mathrm{CT_{st}}$ are the stationary concentrations of LE and CT states, respectively, $\sigma_{\mathrm{PL,LE}}(\omega_{pr})$and $\sigma_{\mathrm{PL,CT}}(\omega_{pr})$are the spectral shapes for emission from LE and CT states, respectively, normalized to unit spectral weight, and r is a geometrical constant comprising outcoupling efficiency and detector geometry. The spectral shape $\sigma_{\mathrm{PL,LE}}(\omega_{pr})$is known from PL/EL spectra of the pure o-IDTBR film/device, while for the spectral shape of $\sigma_{\mathrm{PL,CT}}(\omega_{pr})$, we assume a Gaussian band with the center at $\mathrm{E_{CT}}$ and a width of 0.08 eV; this is chosen to be only slightly larger than the width of the LE emission (fitted to 0.063 eV) reflecting the strong LE-CT hybridization which should yield similar spectral shapes albeit shifted in energy.

(3) Finally, the rise of CS states is modeled by letting $g_{pe} = \delta(0)$, a delta pulse with unity intensity at time zero, and solving the dynamics of LE, CT, and CS states by diagonalizing the transfer matrix. Since no stationary charge density is created by this experiment, we do not consider charge recombination ($\mathrm{k_L}$=$\mathrm{k_{CS,CT}}$=0).

Multi-objective optimization is performed using linear scalarization of the offsets of the simulated targets with respect to the experimental targets: (1) an IQE of 85%, (2) the root mean square error between the actual EL spectrum and the simulated one, and (3) the deviation of the CS rise time from 40 ps.

The forward rate constant for charge separation is given by

$$k_{CT,CS} = k^0_{CT,CS} \cdot exp(-AE_{CT,CS}), \qquad \text{(S11)}$$

$AE_{CT,CS}$ being a formal activation energy in the Marcus picture, and $k^0_{CT,CS} = 10^{13} s^{-1}$ the prefactor ("attempt-to-escape frequency"). The backward rate constants are obtained from the forward rate constants by considering the two Boltzmann equilibria:

$$k_{CT,LE} = k_{LE,CT} \cdot D_{LE}/D_{CT} \cdot exp(E_{CT} - E_{LE}), \quad \text{(S12)}$$

$$k_L = k_{L,0} \cdot D_{CT}/D_{CS} \cdot exp(-(\Delta E_{CT,CS} + AE_{CT,CS})/k_B T), \quad \text{(S13)}$$

where D_{LE}, D_{CT} and D_{CS} are the degeneracy factors of LE, CT, and CS states, respectively, k_B is Boltzmann's constant, and T is the absolute temperature. The constant $k_{L,0}$ is given by standard Langevin-type recombination:

$$k_{L,0} = q(\mu_n + \mu_n)/(\varepsilon_0 \varepsilon_r), \quad \text{(S14)}$$

where q is the elementary charge, μ_n and μ_p are the electron and hole mobilities, respectively, and ε_0 is the dielectric constant in vacuum and ε_r is the relative dielectric constant. Assuming μ_n.= μ_p = 10^{-4} cm^2/Vs and ε_r=3, we obtain $k_{L,0}$ = 1.2.10^{-10} cm^3/s. In the multi-objective optimization, we allowed k_{L0} to float around this value. We found that for $AE_{CT,CS}$ < 0.1, $k_{L,0}$ tended towards the upper limit, otherwise towards the lower limit of the floating range, see Supplementary Figure 13,c. This means that we obtain quantitative values for $k_{L,0}$ and $AE_{CT,CS}$ at the same time. However, as Supplementary Figure 13 shows, we still can reach a general conclusion on the influence of the CT energy on the maximum achievable PCE.

The optimization is performed by varying $\Delta E_{LE,CT}$, $AE_{CT,CS}$, k_{L0} and k_{extr}. We fixed the forward constant $k_{LE,CT}$ = 10^{11} s^{-1} because it has been shown that the precise value of $k_{LE,CT}$ does not play a significant role for the resulting dynamics as long as it is faster

than 100 ps, a condition safely met in high performance D-A blends [Classen et al., Nature Energy 2020].

From the measured EQE values (exceeding 80%), we conclude IQE values above 90% which means that the interface can be reached by excitons formed almost anywhere inside the bulk. Due to the stochastic nature of diffusion (as distinguished from a drift), it follows that also an LE state produced at the interface via the LE ⇔ CT equilibrium, can reach any point inside the bulk. We therefore conjecture that detailed balance is indeed established in our experiments, which allows us to take diffusion implicitly into account by introducing a Boltzmann degeneracy factor ratio D_{CT}/D_{LE} referring to a ratio of interfacial and bulk states.

In Supplementary Table 3, we summarize the fixed parameters and their provenience. Here, we comment on the degeneration ratios from reference(iv): From the measured EQE values (exceeding 80%), we conclude that the IQE values are above 90%, which means that the interface can be reached by excitons formed almost anywhere inside the bulk. Due to the stochastic nature of diffusion (as distinguished from a drift), it follows that also an LE state produced at the interface via the LE ⇔ CT equilibrium, can reach any point inside the bulk. We therefore conjecture that detailed balance is indeed established in our experiments, which allows us to take diffusion implicitly into account by introducing a Boltzmann degeneracy factor gLE,CT referring to a ratio of interfacial and bulk states.

Supplementary Table 3. Parameters used for the multi-objective optimization.

E_{LE}	E_{CS}	k_r	k_{nr}	$k_{r,ct}$	$k_{nr,ct}$	D_{CT}/D_{LE}	D_{CS}/D_{CT}
1.65 eV[iv]	1.44 eV[iv]	$1.2 \cdot 10^6$ s^{-1} [iv]	$1.9 \cdot 10^9$ s^{-1} [iv]	$3 \cdot 10^5$ s^{-1} [iv]	$2.8 \cdot 10^{10}$ [iv]	0.1 [iv]	30

To get more insight into the optimization procedure, we do not blindly fit all 4 free parameters at once. Instead, we vary two of them ($\Delta E_{\mathrm{LE},CT}$and $AE_{CT,CS}$) in a grid, fitting the remaining two parameters for each grid point.

In Supplementary Figure 13, we present the result of the multi-objective optimization. In the grid, we have varied $\Delta E_{\mathrm{LE},CT}$from -0.3 to 0.3 eV, and $AE_{CT,CS}$from 0 to 0.2 eV. For each grid point, kextr and kL0 was optimized to minimize the Euklidean distance (RMSE) of the three objectives to the perfect match. As can be seen in Supplementary Figure 13, the lowest Euklidean distance is obtained along a valley that starts at a negative driving force of -0.08 eV (as negative activation energies do not exist) and ends at a positive driving force of around 0.03 eVwith a soft minimum at about -0.01 eV. The best match of the three objectives is therefore obtained for a nearly vanishing driving force for exciton splitting. This can be understood by considering the fast nonradiative decay of the CT state in WF3-OIDTBR, as taken from ref [Classen NatEnergy]. If the overall CT lifetime is shorter than the LE lifetime, then the high experimental IQE values can only be explained by an equilibrium far on the LE side. On one hand, this explains the perfect agreement between the EL spectra of the blend and the pure acceptor; on the other hand it poses a challenge to model the CS rise time. The more we assume the equilibrium on the LE side (by virtue of negative $\Delta E_{LE,CT}$), the lower the activation energy $AE_{CT,CS}$ for charge separation must be to still explain the 40

ps rise time of the CS state. Therefore we obtain a diagonal shape of the valley, marked in orange, where the 3 objectives match best. Due to the considerable extension of this diagonal valley, we can only give an upper limit to the driving force for exciton splitting, which is at about 0.05 eV.

In Supplementary Figure 14a, we display the deviation of the 3 individual objectives from the experimental values, for the region close to global minimum, shown in Fig. 14b. A clear Pareto front can be observed, which means that the system is underdefined: with the given model, and the given degrees of freedom, we cannot match all 3 objectives at the same time. For example, it is possible to perfectly match IQE and the charge separation time, but on the cost of an imperfect match of the EL spectrum, now showing too much CT contribution. Vice versa, we are able to perfectly match the EL spectrum and IQE, but then the charge risetime becomes too slow.

The occurrence of a pareto front clearly shows that the system that we are using, is not over-defined. It is in principle possible to add another degree of freedom, in order to achieve a perfect match of all three objectives.

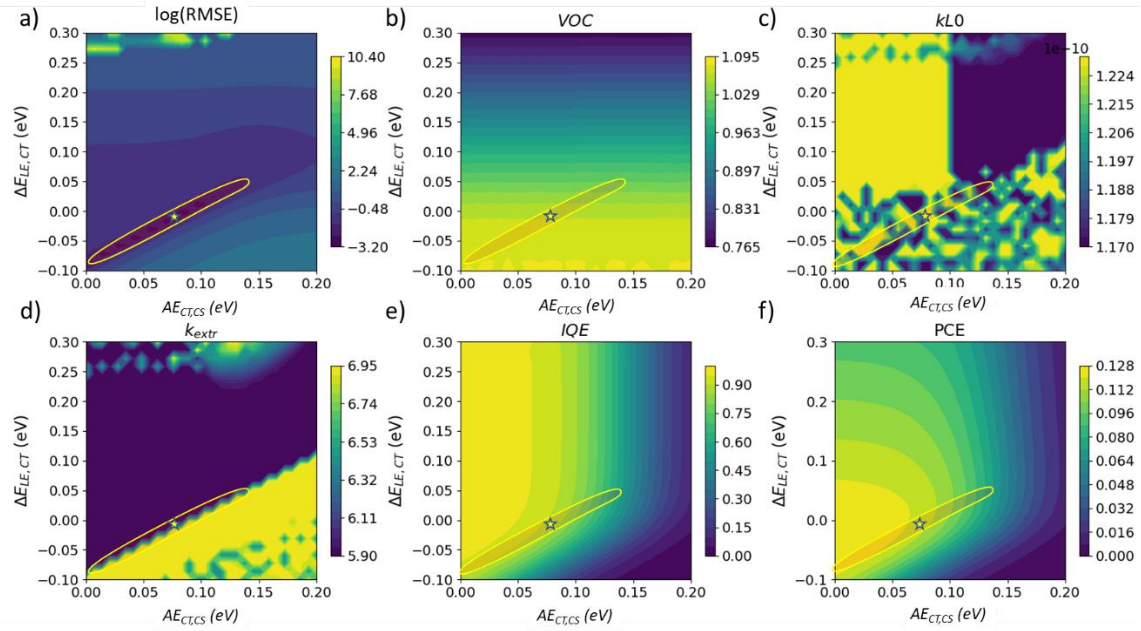

Supplementary Figure 13. Result of the multi-objective optimization of the three objectives using the rate equation scheme (S17 – S19). Upper row (a-c), from left: RMSE – the root mean square error of all objectives, that is, the Euklidean distance to the perfect match, here shown on a logarithmic false color scale, as function of the driving force for exciton splitting and the activation energy for charge separation. VOC – the open circuit voltage, calculated using the simple Langevin model, shifted by -0.23V to match the experimentally determined VOC=1.08 V at the optimum position, marked as a red cross. k_{L0} – the Langevin recombination constant (bimolecular encounter rate of opposite charges). Lower row (d-f), from left: k_{extr} – extraction rate constant; IQE – internal quantum efficiency; PCE, the predicted power conversion efficiency, using the predicted VOC and IQE and the experimental FF=0.68.

In Supplementary Figure 13, we also show the predicted VOC, as calculated from the simple disorder-free Langevin model [James C. Blakesley* and Dieter Neher, PHYSICAL REVIEW B 84, 075210 (2011)]. As expected, VOC does not depend on

the activation energy, as the value of the latter only influences the time of installation of the CT ↔ CS equilibrium, not the position of the equilibrium itself, which controls the stationary CS density and thus VOC. Also as expected, it depends on the energy of the CT or LE state, whichever is lower, which can be seen by the fact that VOC depends on the driving force as long as it is positive but then levels off at negative driving forces. Note that due to ignoring disorder effects [Neher 2011] the predicted VOC at the position of the best fit (marked by a red cross) is predicted too high (1.2 V instead of the experimentally determined 1.08 V). Therefore, we downshifted the whole graph by 0.23 eV, which is equivalent with assuming a disorder-induced effective bandgap [Neher 2011]. Multiplying this empirically corrected VOC with IQE, the absorbed solar flux and the experimental FF=0.68, and dividing by the incident solar flux, we obtain PCE as shown in Supplementary Figure 13f. Obviously, since we corrected the calculated VOC by an empirical term, it is no surprise that also the experimental PCE=12.2% is correctly reproduced at the optimum position. However, the important point is that this value is close to the optimum achievable value for a blend of this composition, namely PCEmax = 12.8%. This notion holds for all combinations of driving forces and activation energies that are able to approximate all three objectives (combinations forming the orange valley in the graph showing RMSE). In summary, from Supplementary Figure 13 it becomes clear that WF3:o-IDTBR forms a D-A interface that places the CT energy very close to the position yielding maximum PCE. From comparing the panels showing VOC, IQE and PCE, it becomes clear that optimum PCE is reached at a CT energy which is a close as possible to a point where the VOC saturates towards lower driving forces, and where the IQE saturates towards higher driving forces. The fact that WF3:o-IDTBR matches this point exactly, explains why this material matches high IQE and low VOC losses at the same time.

The method that we used, is general for BHJ blends and can be easily adapted to other materials if the specific parameters for LE and CT decay are known.

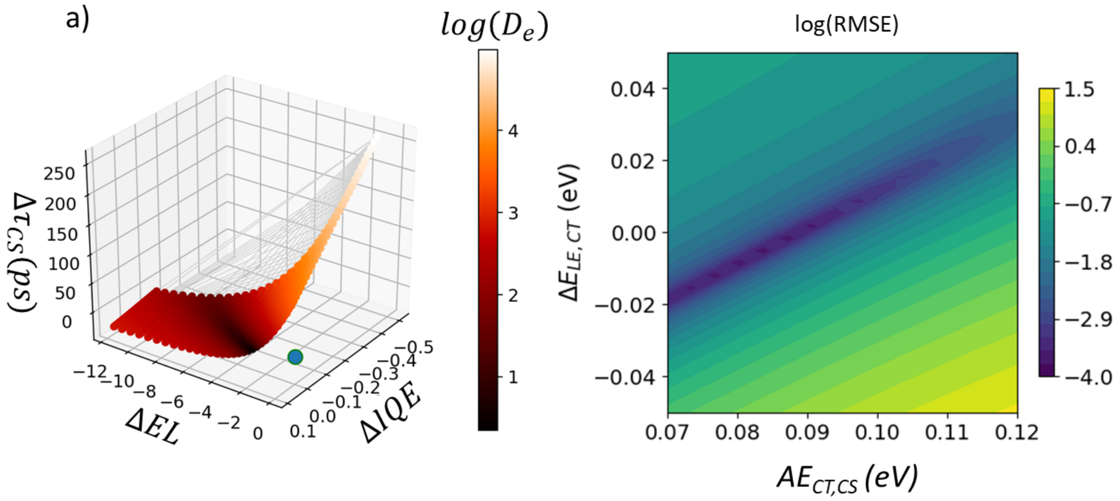

Supplementary Figure 14. Detailed multiobjective optimization around the global minimum. a) Deviation of the three objectives for each fitted grid point, showing a clear Pareto front inhibiting perfect match (identified by the green-blue circle). The Euklidean distance .D_e is shown on a logarithmic color scale

SUPPLEMENTARY NOTE 4: Quantum-Chemical calculations

To further analyze the photophysical properties of the WF3:O-IDTBR blend we carried out (time-dependent) density-functional theory (DFT) calculations using the B3LYP hybrid functional[vi vii] which is known to describe very reliably ground state properties of organic molecules. These calculations were performed using the TURBOMOLE software package (Version 7.1.1)[viii], the def2-TZVP[ix] basis set and the Grimme D3[x] dispersion correction to account for long-range van-der-Waals interactions in the

WF3:O-IDTBR dimers. In a first step, we separately optimized the geometries of the O-IDTBR molecule and of one repeat unit of the WF3 polymer, saturated by hydrogen. Next we carried out geometry optimizations for WF3:O-IDTBR dimers. Depending on the starting geometry different dimer arrangements were found which all represent minima on the potential energy surface. This should reflect the actual situation at the WF3:O-IDTBR interface where various slightly different arrangements can be expected to be present. In the following results obtained with the energetically lowest dimer will be discussed. However, calculations for two more considered arrangements give similar results, see Tables T5 and T6 and Supplementary Figs. 16 and 17, showing that the conclusions drawn from the DFT calculations do not depend significantly on details of the dimer geometry.

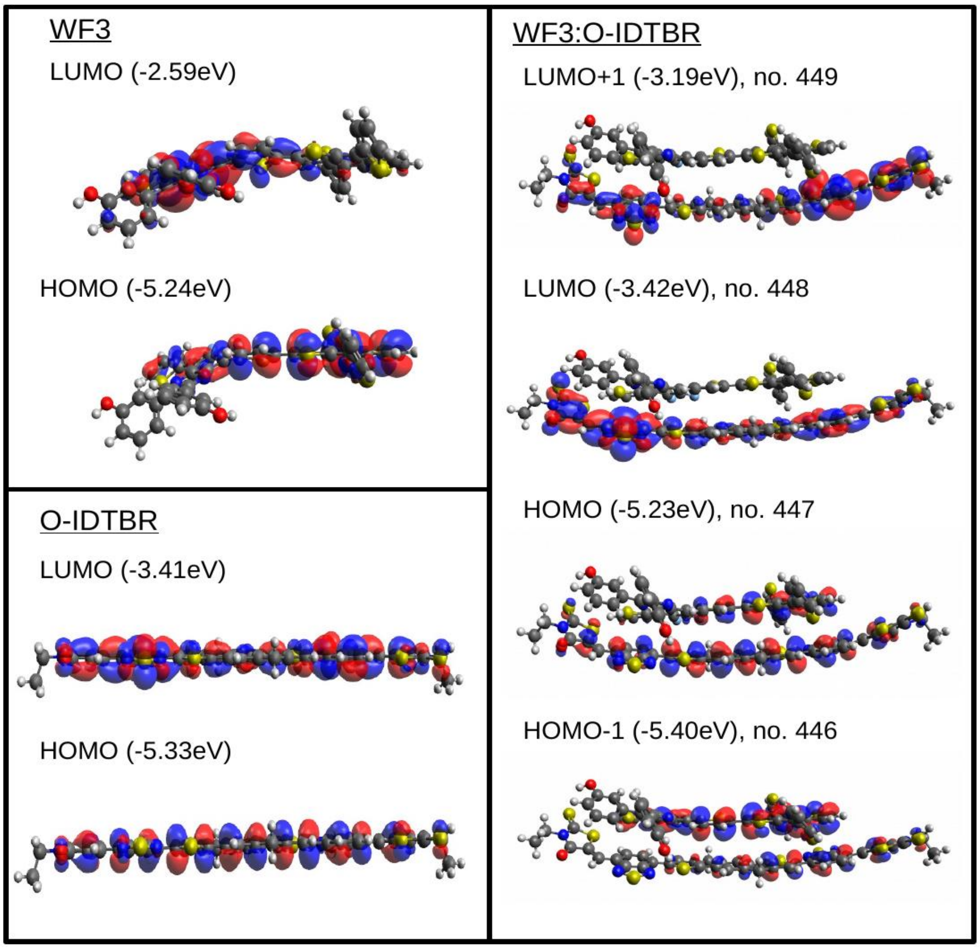

Supplementary Figure 15: Frontier orbitals of WF3, O-IDTBR and the interacting dimer; contour surfaces for an iso-value of 0.02 a.u. are displayed.

The left panels of Supplementary Fig. 15 show the highest occupied molecular orbital (HOMO) and the lowest unoccupied molecular orbital (LUMO) of the WF3 and the O-IDTBR monomer and their energies, respectively. An important observation is that the HOMO energies of both monomers differ by only 0.1 eV. This close alignment of the HOMO energies of donor and acceptor is a peculiarity of the WF3:O-IDTBR system with far reaching consequences. In Kohn-Sham or generalized Kohn-Sham calculations the HOMO eigenvalue formally equals the negative of the ionization potential (IP). In practice, due to the inevitable approximations in the employed exchange-correlation potentials, HOMO eigenvalues typically yield only quite inaccurate estimates for IPs. Therefore we did additional calculations for the WF3 and O-IDTBR cation, both in the geometry of the neutral species and with geometry optimization of the cation and subsequently calculated the ionization potentials by total energy differences, which is more reliable. The resulting vertical and adiabatic IPs are listed in Supplementary Table 2. Again, this confirms the close level alignment of donor and acceptor.

	IP vertical [eV]	IP adiabatic [eV]
WF3	6.20	6.06
O-IDTBR	6.15	6.07
WF3:O-IDTBR dimer	5.96	5.78

Supplementary Table 2: Vertical and adiabatic ionization potentials of WF3 and O-IDTBR and WF3:O-IDTBR

In the right panel of Supplementary Fig. 15 the energetically highest two occupied and the energetically lowest two unoccupied orbitals are displayed together with their eigenvalues. What can be seen is that the HOMOs of the two monomers strongly interact in the dimer. The HOMO and the HOMO-1 both are located on both constituents of the dimer. In fact, optical inspection suggests that the HOMO has somewhat more acceptor and the HOMO-1 somewhat more donor character. To test the robustness of this finding we calculated the cation of the dimer, and found very similar orbitals, see Supplementary Fig. 16, and an IP of 5.78 eV, which is somewhat lower than that of the monomers.

Next, we carried out time-dependent DFT (TDDFT) calculations to characterize the relevant optical excitations. TDDFT excitation energies typically exhibit a strong dependence on the choice of the used exchange-correlation functional and generally are not highly accurate. Indeed, semilocal functionals cannot even qualitatively describe CT excitations. With hybrid functionals CT excitations are accessible, however, the amount of non-local exchange in the functional has a significant effect on the excitation energies, particularly on those with CT character. One possibility to deal with this situation is to individually tune the amount of nonlocal exchange for each molecule.[xi] Because we want to treat monomers as well as dimers with the same density functional we did not do this. Instead we used in all cases the B3LYP functional, which is known to yield excitation energies of reasonable quality for organic molecules of the type considered here.

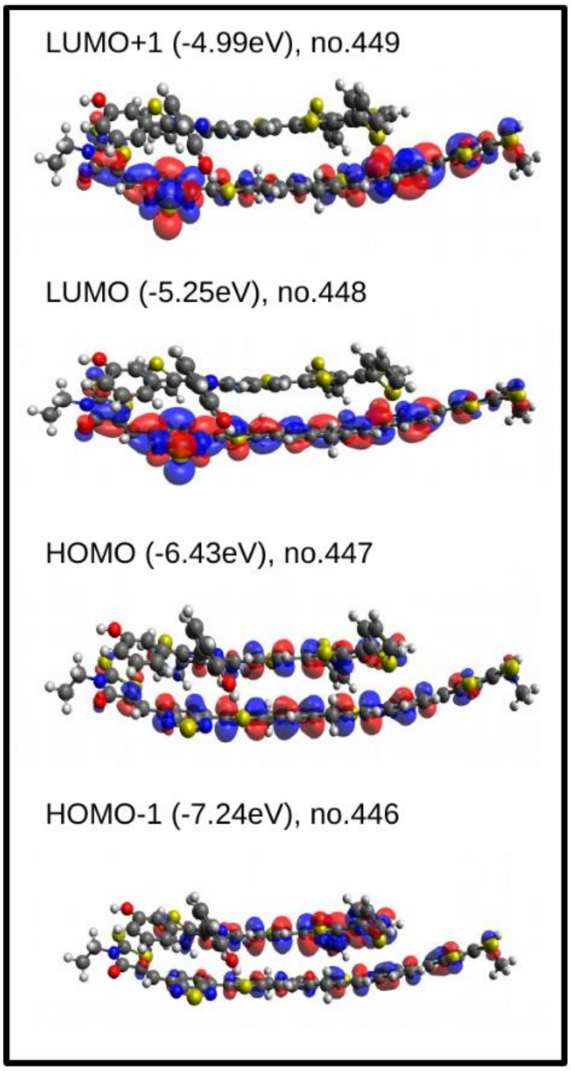

Supplementary Figure 16: Frontier orbitals of the positively charged WF3:O-IDTBR dimer; contour surfaces for an iso-value of 0.02 a.u. are displayed

	Excitation Energy [nm / eV]	Oscillator Strength	Orbital contributions
WF3	461.79 / 2.69	1.60E-001	HOMO-1 → LUMO (95.4%)

	539.64 / 2.30	4.63E-001	HOMO → LUMO (96.7%)
O-IDTBR	636.70 / 1.95	5.27E-006	HOMO → LUMO+1 (95.0%)
	728.33 / 1.70	2.34E+000	HOMO → LUMO (98.2%)
O-IDTBR (1. Ex. (2. Opt.)	791.02 / 1.57	2.51E+000	HOMO → LUMO (98.2%)

Supplementary Table 3: Vertical excitations of WF3 and O-IDTBR monomers

In Supplementary Table 3 the energetically lowest excitation energies of the individual monomers are listed. The calculated vertical HOMO LUMO excitation energy of the acceptor, O-IDTBR, with 1.70 eV agrees very well with the experimental findings. From a geometry optimization of the first excited state an adiabatic excitation energy of 1.57 eV is obtained. The calculated Stokes shift of 0.13 eV agrees well with the experimental estimate of about 0.1 eV.

Excitation Energy [nm / eV]	Oscillator Strength	Orbital contributions
652.05 / 1.90	1.61E-001	446 → 449 (50.6%)
		447 → 449 (31.5%)

		445 → 448 (13.1%)
712.03 / 1.74	3.66E-003	447 → 449 (61.4%)
		446 → 449 (24.5%)
743.82 / 1.67	7.23E-001	446 → 448 (82.2%)
		447 → 448 (10.6%)
807.51 / 1.54	6.39E-001	447 → 448 (85.5%)
		446 → 448 (8.2%)

Supplementary Table 4: Vertical excitations of the interacting WF3:O-IDTBR dimer; HOMO: 447, LUMO: 448

Supplementary Table 3 considers the energetically lowest four excitation energies of the WF3:O-IDTBR dimer. The energetically lowest two excitations are excitations from the HOMO and the HOMO-1 of the dimer into the LUMO which is located at the acceptor, see Supplementary Fig. 15. Usually these excitations would be the CT excitation from the HOMO of the donor unit in the dimer to the LUMO of the acceptor unit in the dimer and the HOMO-LUMO excitation on the acceptor unit. Here, however, the HOMO and HOMO-1 of the dimer are both located on both monomer units. Moreover, the HOMO-LUMO excitation of the dimer has some contribution from the HOMO-1, LUMO excitation and vice versa, see Supplementary Table 3. In order to further characterize the two excitations, we calculated the difference electron densities upon excitation. These are displayed in Supplementary Fig. 17.

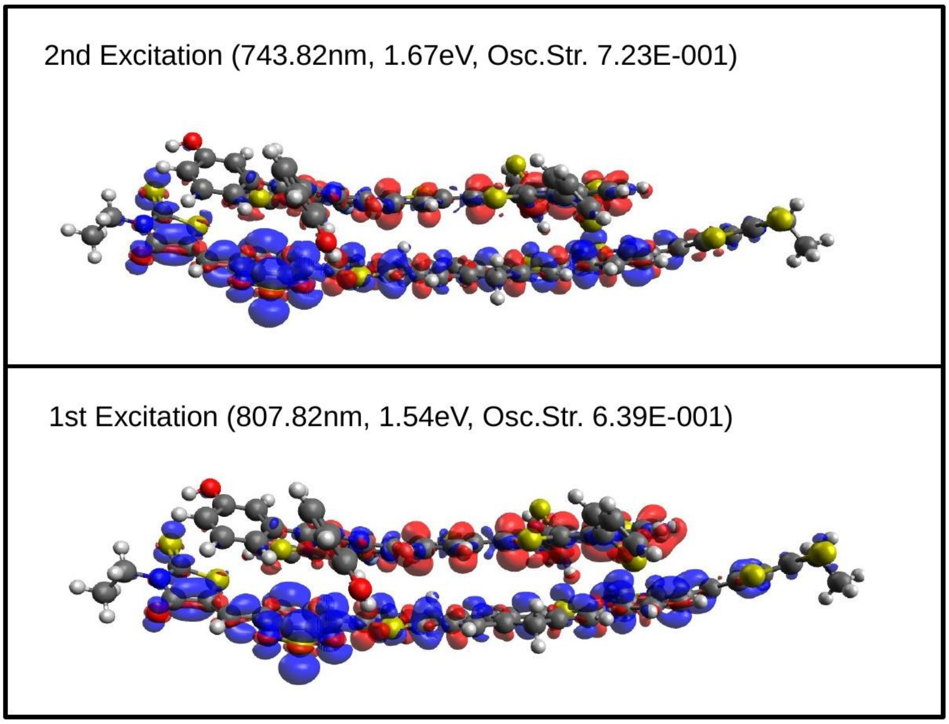

Supplementary Figure 17: Difference densities of the WF3:O-IDTBR dimer of the lowest two vertical excitations; contour surfaces of an iso-value of 0.0005 a.u. are displayed; red: density depletion, blue: density accumulation

It can be seen that both excitations have some CT character but additionally to some extend are HOMO-LUMO excitations on the acceptor. This is corroborated by the oscillator strengths, both excitation have significant oscillator strength and thus are not pure CT excitations, like, e.g. the third excitation, see Supplementary Table 3. On the other hand, the oscillator strengths of both excitations are by a factor of roughly 3 smaller than that of the HOMO-LUMO excitation on the individual acceptor, see Supplementary Table 3. From optical inspection of the charge difference densities of Supplementary Table 3 the first excitation seems to have somewhat more CT character than the second excitation which is closer to an excitation on the acceptor unit. The energy difference between the two excitations is only 0.13 eV. Thus if one would

consider the first excitation as the CT excitation and the second as the HOMO-LUMO excitation on the donor unit, hole transfer at the interface is driven by 0.13 eV. Compared to the HOMO-LUMO excitation energy on the individual O-IDTBR acceptor molecule of 1.70 eV (Supplementary Table 3) the first excitation energy on the dimer with 1.54 eV (Supplementary Table 4) differs by 0.16 eV, which again is very small. A full description of the exciton dynamics is beyond the scope of this work. However, the findings that the HOMO orbitals of donor and acceptor strongly mix in the dimer and that the first two excitation both have CT character and simultaneously have contributions from the HOMO-LUMO excitation on the acceptor unit show that the HOMO levels of donor and acceptor not only are energetically aligned but additionally interact strongly which might facilitate a conversion from one state into the other state.

WF3/O-IDTBR dimers	Formation energy [eV]	Formation energy [kcal/mol]
Geometry 1	-2.14	-49.26
Geometry 2	-2.12	-48.77
Geometry 3	-1.98	-45.55

Supplementary Table 5: Formation energies of the different WF3/O-IDTBR dimer geometries

For the WF3:O-IDTBR dimer we found two geometries with a similar amount of formation energy and one less stable geometry. Formation energies were calculated as energy difference between the dimer energy and the sum of the monomer energies.

The vertical excitation properties do not depend significantly on the geometry of the dimer as the hybridization of the excitations occurs in all considered geometries with

similar oscillator strengths. The energy difference between the lowest two excitations, which we consider as hole transfer barrier, does also not differ significantly, see Supplementary Tables 6 and 7.

Excitation Energy [nm / eV]	Oscillator Strength	Orbital contributions
653.19 / 1.90	1.15E-001	446 → 449 (53.3%)
		447 → 449 (28.8%)
		445 → 448 (12.9%)
708.35 / 1.75	6.34E-003	447 → 449 (61.8%)
		446 → 449 (21.5%)
738.86 / 1.68	6.94E-001	446 → 448 (86.4%)
		447 → 448 (10.6%)
803.03 / 1.54	6.91E-001	447 → 448 (86.4%)
		446 → 448 (8.2%)

Supplementary Table 6: Vertical excitations of the interacting WF3:O-IDTBR dimer (Geometry 2); HOMO: 447, LUMO: 448

Excitation Energy [nm / eV]	Oscillator Strength	Orbital contributions

665.24 / 1.86	5.37E-002	446 → 449 (63.1%)
		447 → 450 (13.5%)
		447 → 449 (13.0%)
747.32 / 1.66	4.61E-001	446 → 448 (44.0%)
		447 → 449 (43.5%)
788.24 / 1.57	4.94E-001	446 → 448 (51.0%)
		447 → 449 (26.7%)
		446 → 449 (13.8%)
857.29 / 1.45	3.61E-001	447 → 448 (85.2%)
		447 → 449 (11.0%)

Supplementary Table 7: Vertical excitations of the interacting WF3:O-IDTBR dimer (Geometry 3); HOMO: 447, LUMO: 448

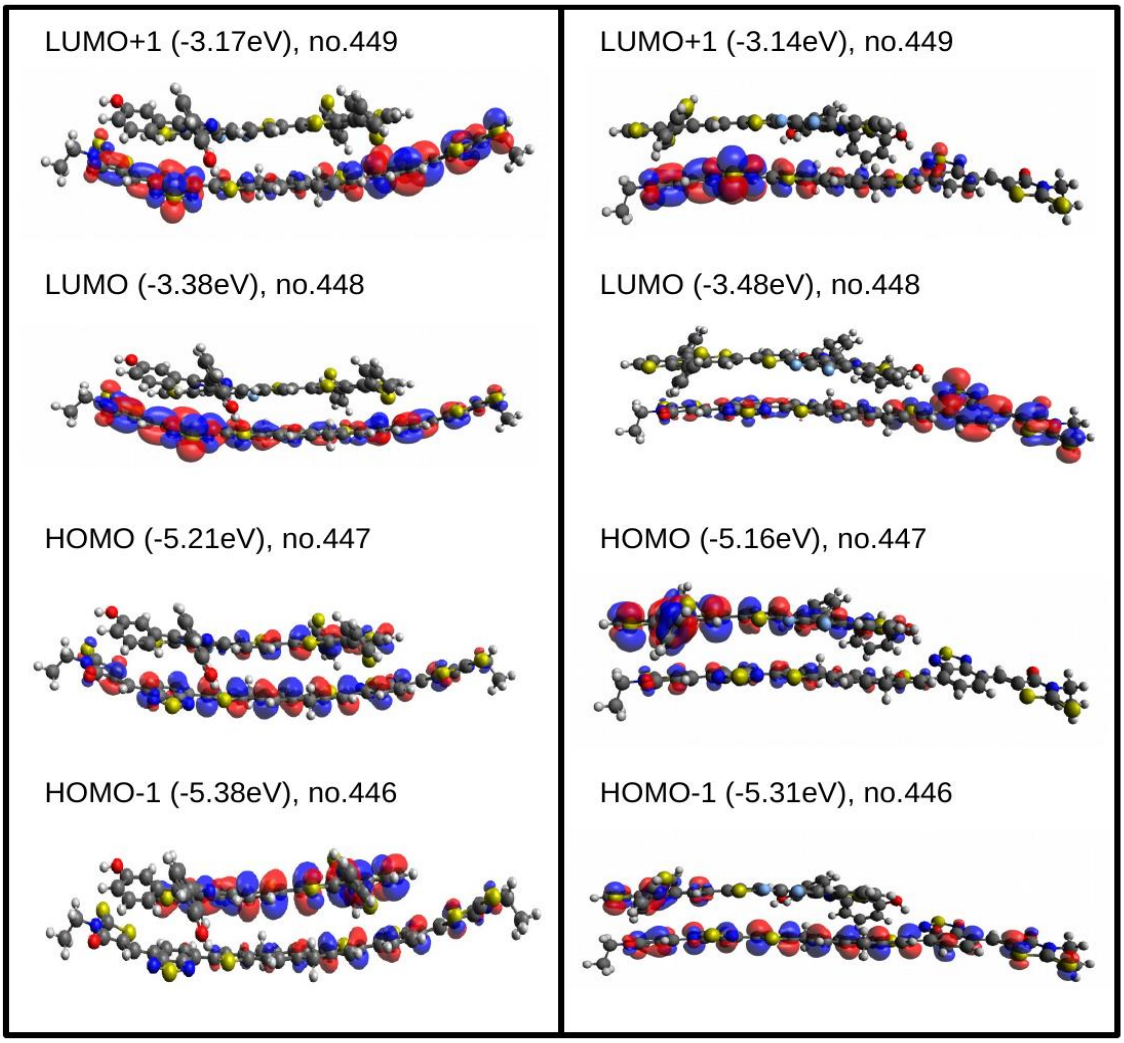

Supplementary Figure 18: Frontier orbitals of different WF3:O-IDTBR dimer geometries, left panel: Geometry 2, right panel: Geometry 3; contour surfaces for an iso-value of 0.02 a.u. are displayed

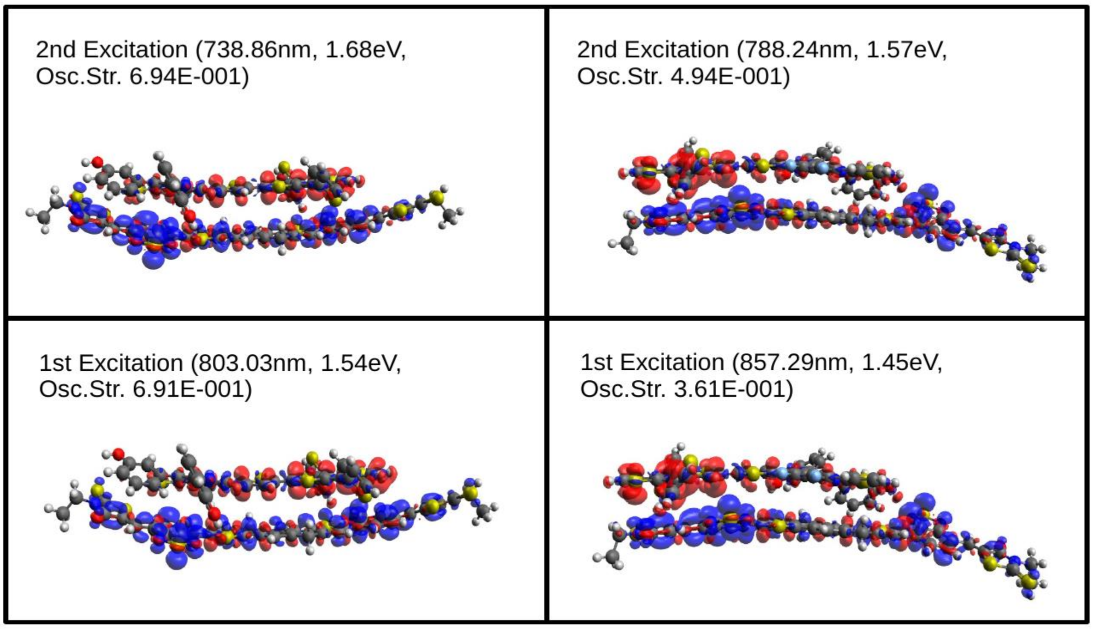

Supplementary Figure 19: Difference densities of the lowest two vertical excitations of the other WF3:O-IDTBR dimer geometries, left panel: Geometry 2, right panel: Geometry 3; contour surfaces of an iso-value of 0.0005 a.u. are displayed; red: density depletion, blue: density accumulation

In summary, the DFT calculations suggest that within the WF3:O-IDTBR system the lowest excitation at the interface is not a pure CT state but somewhat mixed with the HOMO-LUMO excitation on the acceptor unit at the interface. Hole transfer from the O-IDTBR monomers to WF3 has a small driving force because the CT state and the lowest excited state on O-IDTBR exhibit little energy difference. Good level alignment and the strong interaction of the involved orbitals facilitate this process.

SUPPLEMENTARY NOTE 5: Electrostatic calculations

In order to interpret the EA/(CT+CS) curve in Figure 2F of the main text, we need to predict the strength of electroabsorption features as function of the electron-hole separation. Scarognella et al have used an electrostatic model to predict the evolution of the local electric field at certain prominent positions close to the charged state in a co-crystal phase.[xii] In our study, we simulated the electrostatic field in a 10 X 10 X 100 (x,y,z) grid of molecules (edge dimensions 1X1X1 nm each). We assume a sharp interface between the donor (z≤50) and the acceptor (z>50) phases. We place a positive change in the donor phase at position [5,5,50] and calculate the electric field for all molecules in the donor phase. Then we take the sum of the absolute values (for the linear Stark effect causing diffusion-induced broadening or second derivative contributions) or the squares (for the quadratic Stark effect causing a red shift an thus a first derivative contribution)[xiii] of the electric field as function of the distance between the positive and the negative charge which we shift along the positive z direction from z=51 (nearest neighbor charge transfer state) to z = 69. The result is shown in Supplementary Fig. 20. We find that both the linear and quadratic Stark effect should monotonously increase with electron-hole separation, see black and red curves respectively. In the case of the quadratic Stark effect (causing first-derivative shapes in TA spectra) the increase of the EA signal is very strong going from a distance d=1 (nearest-neighbor CT state) to d=2 (next nearest neighbor state with one neutral D molecule in the middle), whereas further increasing d leads only to a weak further increase in EA. Therefore, the experimentally determined quantity EA/(CT+CS), the time-resolved EA signal normalized to the time resolved charge density, is a probe which is particularly sensitive to the first jump away from the interface.

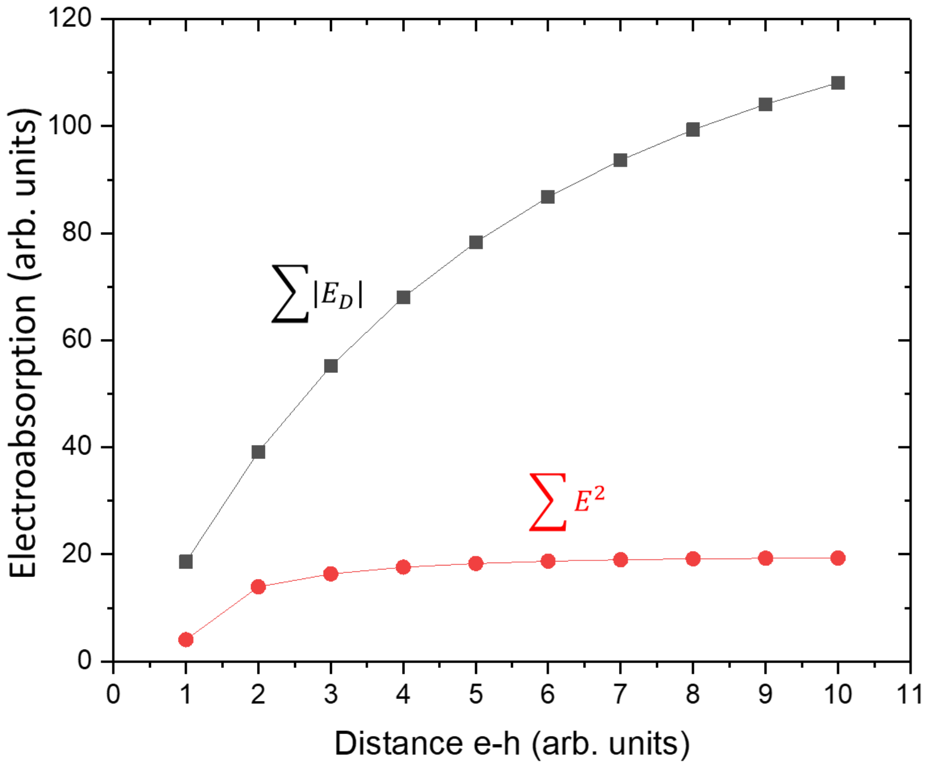

Supplementary Figure 20. Relative strength of electroabsorption signal, expressed as sum over all local field strengths for all molecules in the acceptor phase, as function of the distance between positive and negative charge. Black: Sum over the absolute field strengths (for second derivative contributions); red: sum over the square of the field strength (for first derivative contribution).

SUPPLEMENTARY NOTE 6: Transient absorption after pumping with narrowband pulses

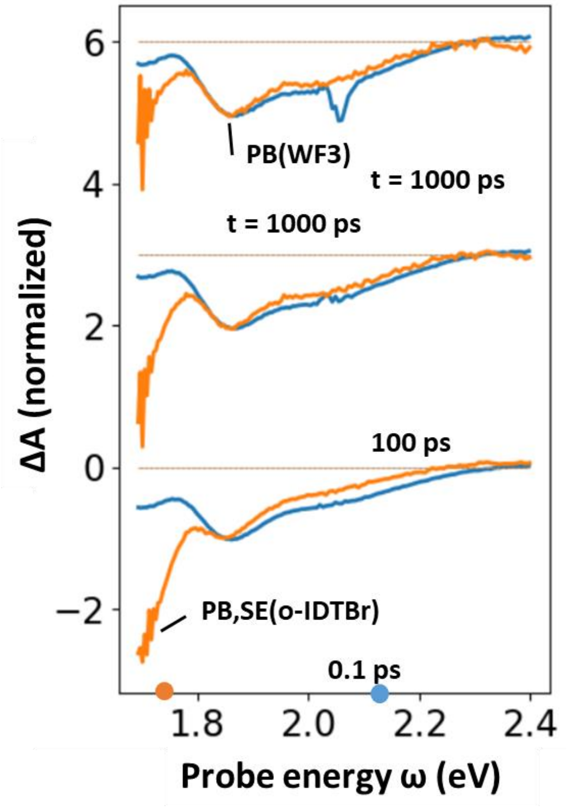

Supplementary Figure 21. Excitation memory of the TA signal after pumping the WF3:O-IDTBR blend with 100 fs narrowband pulses at 1.7 eV and 2.07 eV (orange and blue curves, respectively, energetic position of pump pulses given as orange and blue dots on the X axis) at specified time delays. The TA spectra are normalized to the value at 1.85 eV.

Our high time resolution broadband TA experiments excited both moieties simultaneously. In order to distinguish charge generation after exciting the WF3 and the O-IDTBR phase, we performed TA spectroscopy with 100 fs narrowband pulses tuned to 2.07 and 1.7 eV, respectively (blue and orange curves, respectively, in Supplementary Fig. 21). The curves are normalized to the value at 1.85 eV,

corresponding to the PB of the WF3 phase. Immediately after pumping, the relative amount of PB(O-IDTBR) at 1.7 eV is strongly dependent on the pumping condition, see lower part of Supplementary Fig. 21. After pumping at 1.7 eV, in resonance with the O-IDTBR* exciton, the corresponding PB is much stronger than that of WF3. After pumping at 2.07 eV, we predominantly excite WF3; in this case, the PB of both WF3 and O-IDTBR phases is observed. This agrees with the broadband experiment (Figure 2c) and can be explained by the ultrafast formation of an interfacial CT state. If we pump the O-IDTBR phase, we observe a strong band at 1.7 eV, indicative of PB/SE of the singlet exciton, from which we conclude that such ultrafast CT formation does not occur after pumping the O-IDTBR phase (orange curves in Supplementary Fig. 21). This was also observed in other small-molecule acceptors and explained by the diffusion of smaller excitonic systems towards the interface. [iv]

Once full charge separation has taken place, we expect the PB ratio to be that of the fully charge separated states, and thus independent of the pumping conditions. Surprisingly, we find a significant “excitation memory effect” in the PB ratios even for a delay time of 1000 ps, compare blue and orange curves in the upper part of Supplementary Fig. 21. One possible explanation for this phenomenon is given by the low HOMO offset of donor and acceptor, allowing holes to dwell in either phase; note that the observation of a PB is specific for the phase but unspecific for the kind of photoexcitation; both excitons and charged states in the same phase causing the same PB. The long excitation memory could be the equilibration of the hole between the WF3 and O-IDTBR phases. Having holes and electrons in the same phase would be an extra contribution to avoid formation of interfacial CT states. Further tests are needed to validate this hypothesis.

SUPPLEMENTARY NOTE 7: Photoluminescence transients

In Supplementary Fig. 22, we show a PL transient at 760 nm (in the center of the O-IDTBR emission) of a WF3:O-IDTBR blend excited at 402 nm (blue dots). The PL spectrum of WF3:O-IDTBR blends contains only contributions from O-IDTBR* excitons. The green curve shows a three-exponential fit convoluted with the instrumental autocorrelation function given as orange dots. Although the PL transient closely follows the instrumental response, a delay can clearly be observed, which is best rendered by a biexponential fit yielding $a_1 = 1$; $\tau_1 = 26$ ps; $a_2 = 0.0095$; $\tau_2 = 670$ ps, see green line. Fixing τ_1 to 35 ps and fitting the other parameters, the fit is still acceptable, see red dashed curve. However, assuming $\tau_1 = 50$ ps, clear systematic deviations can be observed, see violet dashed line. In all cases, contribution from the second exponential is very low. Therefore, we state that exciton decay follows a monoexponential decay with less than 50 ps lifetime.

This finding agrees with our numerical modelling (Supplementary Fig. 12), showing the absence of a tailing and therefore demonstrating that the primary equilibrium LE* ⇔ CT, which becomes visible under injection conditions (EL spectra of blend identical to EL spectra of pure acceptor), is rapidly depleted by charge separation (CT ⇔ CS) under open circuit conditions.

Since LE* decay is monoexponential with less than 50 ps lifetime, the residual LE* concentration after 100 ps is below the noise level of a TA experiment. This enables us to use TA spectra for $t > 100$ ps to determine the spectral model for charged states (CT+CS), see Supplementary Fig. 9 and associated text passages.

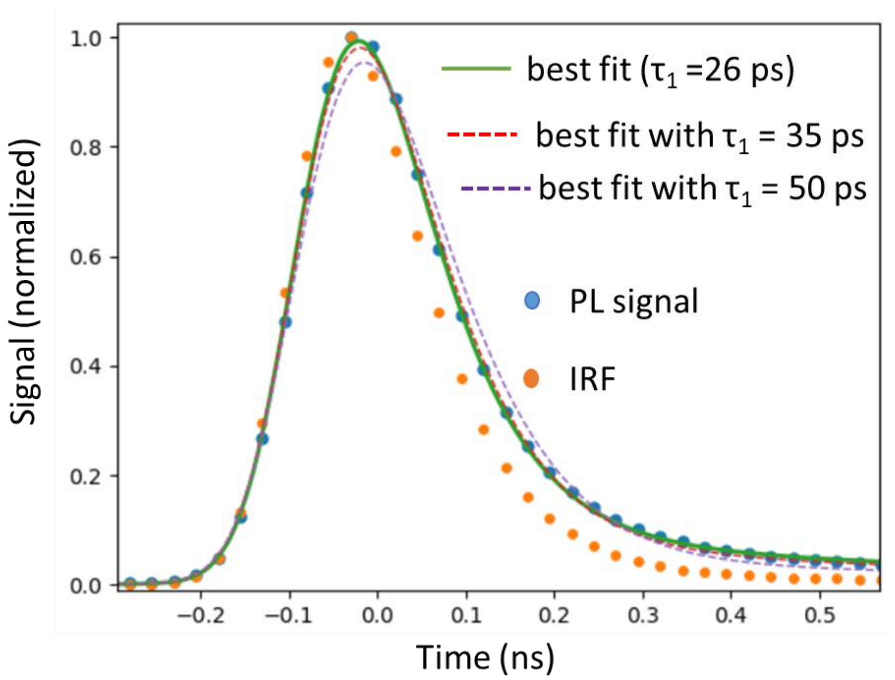

Supplementary Figure 22. Upper panel: PL transient of WF3:O-IDTBR (blue dots) at 760 nm after excitation at 402 nm. The instrumental response function (IRF; light source + detector) is given as yellow dots. Green curve: best biexponential fit, yielding $a_1 = 1$; $\tau_1 = 26$ ps; $a_2 = 0.0095$; τ_2=670 ps. Red dashed curve: best biexponential fit fixing τ_1 at 35 ps; violet dashed curve: best biexponential fit fixing τ_1 at 50 ps.

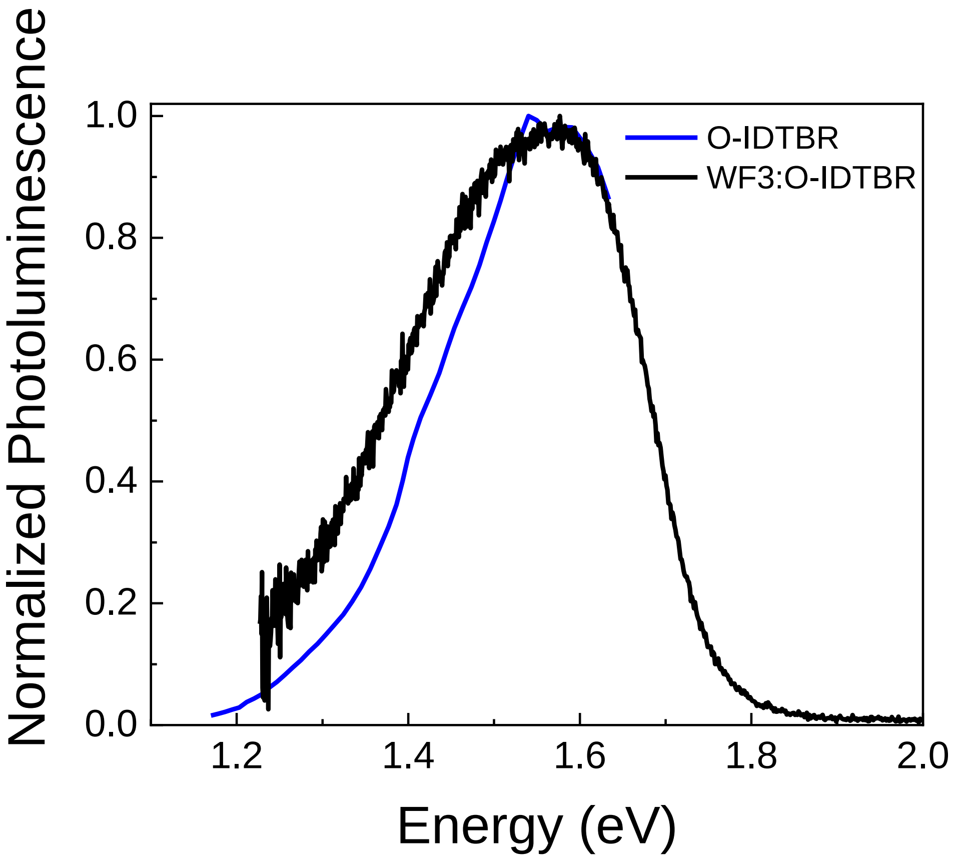

Supplementary Figure 23. Steady state photoluminescence spectra of the blends and pristine annealed O-IDTBR.

SUPPLEMENTARY NOTE 8: Organic solar cells fabrication and characterizations

Materials: WF3 and O-IDTBR were synthetized as reported elsewhere.

Fabrication of Photovoltaic devices: Pre-structured indium tin oxide (ITO) substrates were cleaned with acetone and isopropyl alcohol in an ultrasonic bath for 10 minutes each. After drying, the substrates were spin-coated with 40 nm of zinc oxide (ZnO) and different active layers based on 20 g L^{-1} in Chlorobenzene (CB). To complete the fabrication of the devices 10 nm of MoOx and 100 nm of Ag were thermally evaporated through a mask (with a 10.4 mm^2 active area opening) under a vacuum of ~$1x10^{-6}$ mbar.

J-V measurements: The J-V characteristics were measured using a source measurement unit from BoTest. Illumination was provided by a solar simulator (Oriel Sol 1A, from Newport) with AM1.5G spectrum at 100 mW/cm^2. UV-VIS absorption was performed on a Lambda 950 spectrophotometer, from Perkin Elmer. EQEs were measured using an integrated system from Enlitech, Taiwan. All the devices were tested in ambient air.

FTPS: FTPS-EQE was carried out using a modified Vertex 70 FTIR spectrometer from Brucker optics, equipped with QTH lamp, quartz beam splitter and external detector. A low noise current amplifier (Femto, DLPCA-200) is used to amplify the photocurrent produced upon illumination of the photovoltaic device with light modulated by the FTIR. The output voltage of the current amplifier is fed back to the external detector port of the FTIR, in order to be able to use the FTIR's software to collect the photocurrent spectrum.

EL: EL measurements were performed by using a chopper and applying a constant current (100mA/cm^2) supplied by an external current/voltage source through the devices which have an active area of 0.104 cm^2. The emitted light then collected by a

monochromator and detected by liquid-nitrogen-cooled InGaAs detector. The spectrum was recorded by a standard lock-in technique. The system was wavelength calibrated.

	V_{oc} (V)	J_{sc} (mA cm^{-2})	FF	PCE (%)
WF3:$PC_{70}BM$	0.80	17.11	0.61	8.39 (8.28±0.06)
WF3:O-IDTBR	1.06	16.94	0.68	12.21 (12.13±0.07)

Supplementary Table 8: Key photovoltaics parameters extracted at 1 sun illumination for WF3:$PC_{70}BM$ and WF3:O-IDTBR solar cells.

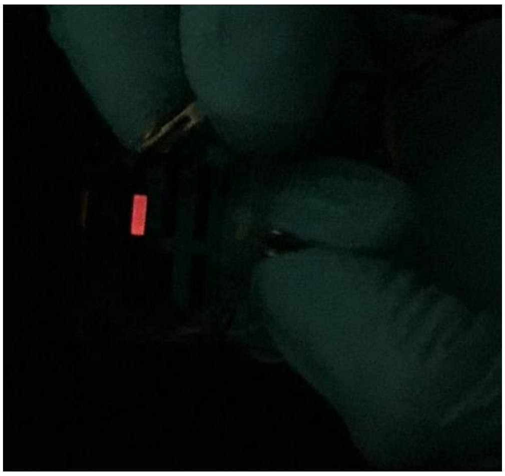

Supplementary Figure 24. Original photograph of the WF3:O-IDTBR electroluminescence.

SUPPLEMENTARY NOTE 9: Estimation of pump-induced relative excitation density

Figure 2D in the main paper shows a significant presence of O-IDTBR excitons 50 fs after the pump pulse. In order to estimate whether these excited states have been produced resonantly by the pump pulse, or by ultrafast energy transfer from resonantly generated WF3 excitons, we estimate here the relative excitation density for both WF3 and O-IDTBR after resonant pumping. To this end, we decompose the ground state absorption spectrum of the WF3-o-IDTBR blend (blue spectrum in Supplementary Figure 25) into individual contributions from WF3 and O-IDTBR (green and red spectra in Supplementary Figure 25, respectively, for details see Supplementary Figure 3). Then we multiply the spectrum of the pump pulse (purple dashed spectrum in Supplementary Figure 25) with either the WF3 or the O-IDTBR spectrum and compare the integral. We find that the pump resonantly generates about 30% O-IDTBR excitons and 70% WF3 excitons. The latter convert instantaneously into WF3-O-IDTBR CT states. Hence, the excited state composition of the TA spectrum of the blend at t=50 fs can be explained by resonant creation of O-IDTBR excitons.

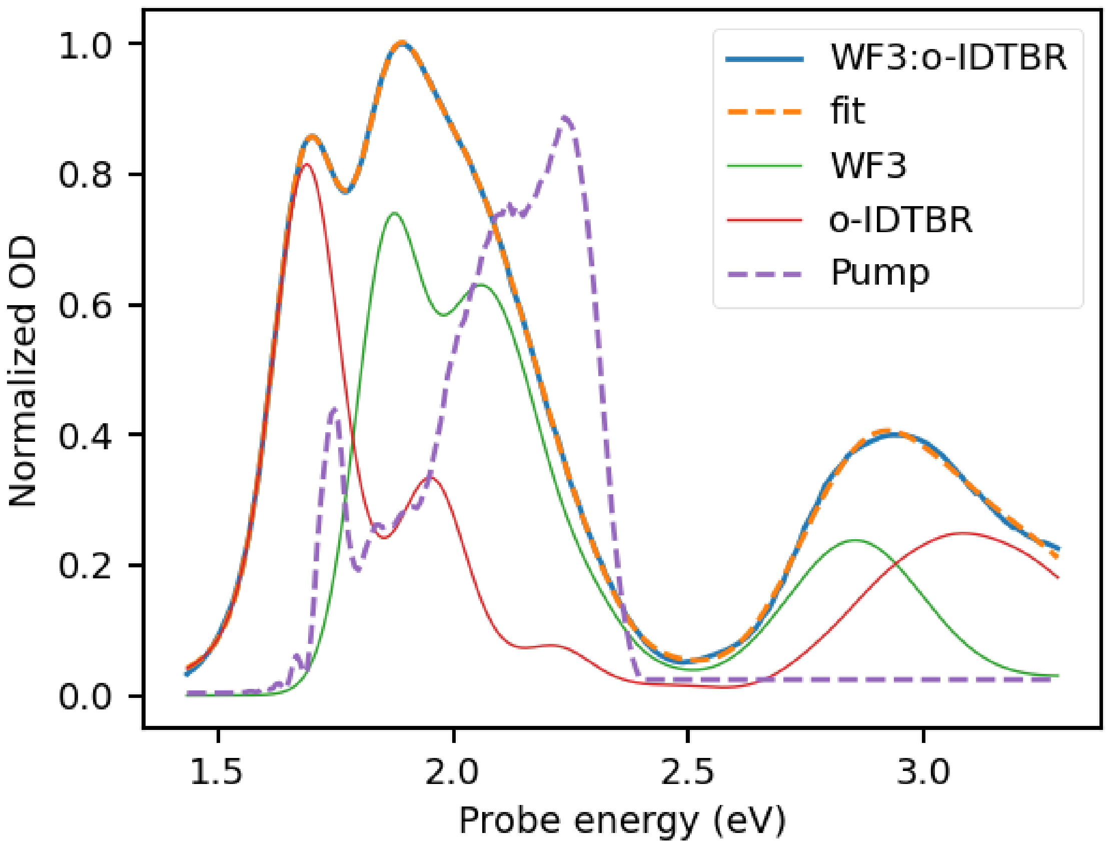

Supplementary Figure 25. Ground state absorption spectrum of WF3-O-IDTBR blend (blue line), and spectral fit (orange dashed line) composed of contributions from WF3 and O-IDTBR (green and red lines, respectively.) The pump pulse is shown as violet dashed line. For details of the spectral decomposition of GA, we refer to Supplementary Figure 3.

SUPPLEMENTARY NOTE 10: Appendix. Procedure of spectral decomposition into contributions from physical states

According to Lambert-Beer's Law, the natural absorption $A = -lnT$, T being the transmission, of N_s electronic states is given by

$$A(\omega, t, J) = \sum_{m=1}^{N_s} \sigma_m(\omega) \cdot s_m(t, J), \quad \text{(SA1)}$$

where $\sigma_m(\omega)$ and $s_m(t, J)$ are the absorption cross-section and the area density of state m, the latter defined as $s_m = c_m/L$, where c_m is the concentration and L is the film thickness. By modulating the pump pulse and measuring A, we can detect the pump-induced changes in A. Defining the difference of a variable X as $\Delta X \equiv X_{pumpon} - X_{pumpoff}, X \in \{A, s\}$, Lambert Beer's Law for the differential absorption yields:

$$\Delta A(\omega, t, J) = \sum_{m=1}^{N_s} \sigma_m(\omega) \cdot \Delta s_m(t, J) \quad \text{(SA2)}$$

Experimentally, ΔA is detected at discrete values for ω_i and t_k, $i \in \{1,2,..,N_\omega\}$, $k \in \{1,2,..,N_t\}$, N_ω and N_t being the number of discrete probe energies and delay times, respectively. After properly removing artifacts (scattering of pump pulse, photoluminescence, chirp of probe pulse), one obtains for each pump intensity a matrix $A^{exp, J}$ whose elements are given by

$$A_{ik}^{exp,J} = \sum_{m=1}^{N_s} \sigma_m(\omega_i) \cdot \Delta s_m(t_k, J) + N_{ik}^J , \quad \text{(SA3)}$$

N_{ik}^Jbeing a stochastic noise contribution to each data point. SA3 can be restated as a matrix equation:

$$A_j^{exp} = \sigma \cdot s_j + N. \quad \text{(SA4)}$$

In SA4, $j \in \{1,2,..N_j\}$ indexes the different pump intensities J_j used, σ is an ($N_\omega \times N_s$) matrix of individual cross-sections of all states, arranged as column vectors, while s_j is an ($N_s \times N_t$) matrix of individual area densities, arranged as row vectors, and N is an ($N_\omega \times N_t$) matrix of measurement noise.

The goal is to solve S4 for s_j, given the experimentally measured set of matrices A_j. To this end, we must reject the noise N and introduce our knowledge about σ:

$$s_j = \sigma^{-1}\left(A_j^{exp} - N\right), \tag{SA5}$$

where σ^{-1} is the pseudo-inverse of σ. In eqns. S3 through S5, we have assumed that σ does not depend on J (only linear absorptions considered), while s_j does. This allows us to concatenate all A_j^{exp} matrices along the time axis, forming one single matrix with elements

$$A_{ir}^{exp}; r = (N_t - 1) \cdot j + k, \tag{SA6}$$

such that

$$A^{exp} = \sigma \cdot s + N. \tag{SA7}$$

where the matrix elements s_{mr} and N_{ir} are given by the same definition of r as in eq. S6. Note that the shape of σ in SA7 is still ($N_\omega \times N_s$), while the shape of s is different from that of s_j, namely ($N_s \times [N_t \times N_j]$). A singular value decomposition (SVD) of A^{exp} yields

$$A^{exp} = U \cdot S \cdot V. \tag{SA8}$$

Here U is a matrix of unitary basis column vectors, V a matrix of unitary row vectors, and S a diagonal matrix of weight coefficients ("singular values"). S can be decomposed into two matrices S_S and S_N containing only those singular values related to signal and noise, respectively, so that $S = S_S + S_N$. Then:

$$A^{exp} = U_S \cdot S_S \cdot V_S + U_N \cdot S_N \cdot V_N = U_S \cdot S_S \cdot V_S + N. \quad \text{(SA9)}$$

In S9, we have exploited the property that a truncated SVD is always optimal in a least squares sense, i.e it solves the equation $N = min([A^{exp} - U_S \cdot S_S \cdot V_S]^2)$; the procedure can therefore be considered a bias free global fitting. In order to distinguish signal from noise related singular values, the autocorrelation function of the corresponding basis vectors can be analyzed; here we simply included the N_{signal} strongest singular values into S_S so that no systematic residuals are observed comparing A^{exp} and $A^{fit} = U_S \cdot S_S \cdot V_S$.

Subtracting SA7 from SA9:

$$\sigma \cdot s = U_S \cdot S_S \cdot V_S. \quad \text{(SA10)}$$

Inserting the identity $R \cdot R^{-1}$into SA10:

$$\sigma \cdot s = U_S \cdot R \cdot R^{-1} \cdot S_S \cdot V_S, \quad \text{(SA11)}$$

and applying the pseudoinverse of $U_S \cdot R$:

$$(U_S \cdot R)^{-1} \cdot \sigma \cdot s = R^{-1} \cdot S_S \cdot V_S, \quad \text{(SA12)}$$

we find that if

$$U_S \cdot R = \sigma, \quad \text{(SA13)}$$

then

$$s = R^{-1} \cdot S_S \cdot V_S. \quad \text{(SA14)}$$

In SA13, R is the rotation-scaling matrix transforming the signal related basis column vectors of the SVD (unitless) into the physically relevant absorption cross-sections (in cm^2) of the available states, while in SA14, the inverse of R transforms the signal related basis row vectors of the SVD into the physically relevant area densities (in cm^{-2})

and thus into the photoexcitation dynamics, our desired quantity. We thus must solve SA13 to find s in SA14.

We solve S13 by assuming physically reasonable test functions for the column vectors in σ, according to the following rules:

- We generally use Voigt or Gaussian band shapes. Vibronic coupling is introduced by assuming one essential vibronic progression with one effective Huang-Rhys factor. The intensity of the (00) vibronic progression can be reduced (H aggregation) or enhanced (J aggregation). In any case, the integral of a full progression is one.
- Thermal population of low-frequency vibrational or torsional modes can be considered by introducing a skewness of the bands.
- All states show a ground state photobleach (PB), which is an inverted and scaled (to area 1) replica of the ground state absorption (GA).
- Emissive singlet excitons show stimulated emission (SE), which is an inverted and scaled (to area 1) replica of the steady state photoluminescence (PL) spectrum.
- Charged states can (but not always) introduce electroabsorption-like (EA) features in molecules in the electronic ground state.

Once we have defined the tests functions in σ, we perform a non-linear optimization of S13 varying the bandshape parameters. During each iteration, optimum values for R are obtained by calculating the pseudo inverse $R = U_S^{-1} \cdot \sigma$, until the residuals $U_S - \sigma \cdot R^{-1}$are minimized. Success of the operation can be shown by calculating s from S14 and then comparing the fitted $A^{fit} = \sigma \cdot s$ to the experimental one, compare thin and bold curves, respectively, in Supplementary Figs. 7c and 10. The following rules are applied:

- All states in the same phase share the same PB, in both shape and intensity. This dramatically reduces the number of free parameters, because the absolute cross-sections and the approximate shape of PB can be derived from GA, because it is the same transition and thus has the same total oscillator strength. Only a small redshift of the center energy and some narrowing (both due to spectral migration effects) is allowed going from GA to PB. A broadening or blue shift is not allowed. A change of total oscillator strength or HR factor is also not allowed.
- For SE, a reduction of the (00) transition with respect to PL is allowed, caused by reabsorption of the typically optically thick films. If strong geometric reorganization in the excited state is expected, also the absolute area of SE can be different from one and therefore different from PB.
- For EA, we assume no spatial correlation of state energies and therefore we allow no spectral migration effects; therefore, exact first and second derivatives of GA are assumed, and no narrowing/redshift is allowed.
- If states have an entirely different electron configuration (neutral => charged; singlet =>triplet), they cannot have exactly the same PA bands.

These physically reasonable restrictions guarantee that a good fit yields correct physical states and at the same time reduces the number of free parameters. After the nonlinear optimization, the thus obtained preliminary rotation matrix should be scaled to the final one such that application of SA14 yields time-resolved concentrations that agree with known boundary conditions, such as the total number of excited states at time zero. If this is not done, the concentrations are not to scale, but still the normalized dynamics are correct. This means that first order rate constants can still be obtained absolutely but not second order (bimolecular) ones. Also, a branching ratio of a parallel reaction of

type A->B; A->0 cannot be given, but the total loss dynamics of A and the formation dynamics of B will still be correct.

For the purpose of the present paper, relative concentrations are sufficient, because we are interested in a change in the population/EA ratio, which we use as a probe for charge separation. Therefore, a scaling of the rotation matrix has not been done, and all concentrations are given in arbitrary units.

Supplementary References

[i] I. H. M. Van Stokkum, D. S. Larsen, R. van Grondelle, *Biochimica et Biophysica Acta*, 2004, **1658**, 82–104.
[ii] J. Shi, A. Isakova, A. Abudulimu, M. van den Berg, O. K. Kwon, A. J. Meixner, S. Y. Park, D. Zhang, J. Gierschner, L. Lüer, Energy Environ. Sci. 2018, 11, 211
[iii] M. Causa et al., Nat Comm. DOI: 10.1038/ncomms12556
[iv]A. Classen et al., Nature Energy 2020, in press
[v]T.M. Burke et al., Adv. En. Mat. 2015 5(11), 1500123
[vi] A.D. Becke, *J.Chem.Phys.,* 1993, **98,** 5648-5652
[vii] C. Lee, W. Yang, R.G. Parr, *Phys.Rev.B,* 1988, **37**, 785-789
[viii] TURBOMOLE V7.1 2016, a development of University of Karlsruhe and Forschungszentrum Karlsruhe GmbH, 1989-2007, TURBOMOLE GmbH, since 2007; available from http://www.turbomole.com.
[ix] A. Schäfer, C. Huber, R. Ahlrichs, *J.Chem.Phys.,* 1994, **100**, 5829-5835
[x] S. Grimme, J. Antony, S. Ehrlich, H. Krieg, *J.Chem.Phys.,* 2010, **132,** 154104
[xi] S. Rafaely-Abramson, S.Sharifzadeh, N. Govind, J. Autschbach, J.B. Neaton, R. Baer, L. Kronik, *Phys.Rev.Lett.* , **2012**, 109, 226405
[xii] M. Scarognella et al., J. Am. Chem. Soc. 2015, 137, 2908−2918

[xiii] P.J.Brown et al., PHYSICAL REVIEW B, VOLUME 63, 125204
