# Supplementary material for: Adjusting the energy of interfacial states in organic photovoltaics for maximum efficiency
Source: Nat Commun. 2021 Mar 19;12:1772. doi: 10.1038/s41467-021-22032-3 (PMC7979693; doi:10.1038/s41467-021-22032-3)
Supplement: Supplementary file 3 — Solar Cells Reporting Summary [file 41467_2021_22032_MOESM3_ESM.pdf]

nature research

Corresponding author(s): Dr Nicola Gasparini

Solar Cells Reporting Summary

Nature Research wishes to improve the reproducibility of the work that we publish. This form is intended for publication with all accepted papers reporting the characterization of photovoltaic devices and provides structure for consistency and transparency in reporting. Some list items might not apply to an individual manuscript, but all fields must be completed for clarity.

For further information on Nature Research policies, including our data availability policy, see Authors & Referees.

Experimental design

Please check: are the following details reported in the manuscript?

1. Dimensions

Item	Yes	No	Details
Area of the tested solar cells	☒	☐	The area of the tested solar cells (10.4 mm2) can be found in the method section
Method used to determine the device area	☒	☐	The device area was determined through an evaporation mask, see methods

2. Current-voltage characterization

Item	Yes	No	Details
Current density-voltage (J-V) plots in both forward and backward direction	☐	☒	In OPV devices the hysteresis effect is not observed.
Voltage scan conditions<br>*For instance: scan direction, speed, dwell times*	☒	☐	Forward scan - 1V/s - dwell = 20 ms
Test environment<br>*For instance: characterization temperature, in air or in glove box*	☒	☐	The devices were characterized in air at room temperature
Protocol for preconditioning of the device before its characterization	☐	☒	No preconditioning are necessary for OPV characterizations.
Stability of the J-V characteristic<br>*Verified with time evolution of the maximum power point or with the photocurrent at maximum power point; see ref. 7 for details.*	☐	☒	The stability of the devices is out of the scope of this work.

3. Hysteresis or any other unusual behaviour

Item	Yes	No	Details
Description of the unusual behaviour observed during the characterization	☐	☒	No hysteresis was observed in our devices.
Related experimental data	☐	☒	N/A

4. Efficiency

Item	Yes	No	Details
External quantum efficiency (EQE) or incident photons to current efficiency (IPCE)	☒	☐	EQE graph in Figure 1d
A comparison between the integrated response under the standard reference spectrum and the response measure under the simulator	☒	☐	The integrated Jsc obtained from the EQE spectra agrees well with the Jsc value obtained from the J–V curves under the simulator within 5% deviation
For tandem solar cells, the bias illumination and bias voltage used for each subcell	☐	☒	N/A

5. Calibration

Item	Yes	No	Details
Light source and reference cell or sensor used for the characterization	☒	☐	Calibrated silicon reference cell - part of the commercial solar simulator
Confirmation that the reference cell was calibrated and certified	☒	☐	By supplier of the solar simulator

Calculation of spectral mismatch between the reference cell and the devices under test	☐ Yes ☒ No	Jsc (solar simulator) and Jsc (via EQE) agree within 5 % error

6. Mask/aperture

Size of the mask/aperture used during testing	☒ Yes ☐ No	Device area is determined by a mask with area of 10.4 mm2.
Variation of the measured short-circuit current density with the mask/aperture area	☒ Yes ☐ No	No variations observed

7. Performance certification

Identity of the independent certification laboratory that confirmed the photovoltaic performance	☐ Yes ☒ No	This work does not report record efficiency, therefore the photovoltaic performance of our devices were not confirmed from independent certification laboratories.
A copy of any certificate(s)<br>*Provide in Supplementary Information*	☐ Yes ☒ No	N/A

8. Statistics

Number of solar cells tested	☒ Yes ☐ No	The average PCE of OSC is obtained from 24 independent devices.
Statistical analysis of the device performance	☒ Yes ☐ No	Statistical results of the devices are shown in Supplementary Table 8.

9. Long-term stability analysis

Type of analysis, bias conditions and environmental conditions<br>*For instance: illumination type, temperature, atmosphere humidity, encapsulation method, preconditioning temperature*	☐ Yes ☒ No	Not relevant for this work. However, shelf life of all devices was excellent.
